# *In vitro* characterization of the antivirulence target of Gram-positive pathogens, peptidoglycan *O*-acetyltransferase A (OatA)

**DOI:** 10.1371/journal.ppat.1006667

**Published:** 2017-10-27

**Authors:** David Sychantha, Carys S. Jones, Dustin J. Little, Patrick J. Moynihan, Howard Robinson, Nicola F. Galley, David I. Roper, Christopher G. Dowson, P. Lynne Howell, Anthony J. Clarke

**Affiliations:** 1 Department of Molecular and Cellular Biology, University of Guelph, Guelph, ON, Canada; 2 Program in Molecular Structure and Function, Research Institute, The Hospital for Sick Children, Toronto ON, Canada; 3 Department of Biochemistry, University of Toronto, 1 King's College Cir, Toronto, ON, Canada; 4 School of Biosciences, University of Birmingham, Edgbaston, Birmingham, United Kingdom; 5 Photon Science Division, Brookhaven National Laboratory, Upton, New York, United States of America; 6 School of Life Sciences, University of Warwick, Coventry, United Kingdom; National Jewish Health, UNITED STATES

## Abstract

The O-acetylation of the essential cell wall polymer peptidoglycan occurs in most Gram-positive bacterial pathogens, including species of *Staphylococcus*, *Streptococcus* and *Enterococcus*. This modification to peptidoglycan protects these pathogens from the lytic action of the lysozymes of innate immunity systems and, as such, is recognized as a virulence factor. The key enzyme involved, peptidoglycan *O*-acetyltransferase A (OatA) represents a particular challenge to biochemical study since it is a membrane associated protein whose substrate is the insoluble peptidoglycan cell wall polymer. OatA is predicted to be bimodular, being comprised of an N-terminal integral membrane domain linked to a C-terminal extracytoplasmic domain. We present herein the first biochemical and kinetic characterization of the C-terminal catalytic domain of OatA from two important human pathogens, *Staphylococcus aureus* and *Streptococcus pneumoniae*. Using both pseudosubstrates and novel biosynthetically-prepared peptidoglycan polymers, we characterized distinct substrate specificities for the two enzymes. In addition, the high resolution crystal structure of the C-terminal domain reveals an SGNH/GDSL-like hydrolase fold with a catalytic triad of amino acids but with a non-canonical oxyanion hole structure. Site-specific replacements confirmed the identity of the catalytic and oxyanion hole residues. A model is presented for the O-acetylation of peptidoglycan whereby the translocation of acetyl groups from a cytoplasmic source across the cytoplasmic membrane is catalyzed by the N-terminal domain of OatA for their transfer to peptidoglycan by its C-terminal domain. This study on the structure-function relationship of OatA provides a molecular and mechanistic understanding of this bacterial resistance mechanism opening the prospect for novel chemotherapeutic exploration to enhance innate immunity protection against Gram-positive pathogens.

## Author summary

Multi-drug resistance amongst important human pathogens, such as methicillin-resistant *Staphylococcus aureus* (MRSA), vancomycin-resistant *Enterococcus* (VRE) and drug-resistant *Streptococcus pneumoniae* (DRSP), continues to challenge clinicians and threaten the lives of infected patients. Of the several approaches being taken to address this serious issue is the development of antagonists that render the bacterial infection more susceptible to the defensive enzymes and proteins of our innate immunity systems. One such target is the enzyme *O-*acetyltransferase A (OatA). This extracellular enzyme modifies the essential bacterial cell wall component peptidoglycan and thereby makes it resistant to the lytic action of lysozyme, our first line of defense against invading pathogens. In this study, we present the first biochemical and structural characterization of OatA. Using both the *S*. *aureus* and *S*. *pneumoniae* enzymes as model systems, we demonstrate that OatA has unique substrate specificities. We also show that the catalytic domain of OatA is a structural homolog of a well-studied superfamily of hydrolases. It uses a catalytic triad of Ser-His-Asp to transfer acetyl groups specifically to the C-6 hydroxyl group of muramoyl residues within peptidoglycan. This information on the structure and function relationship of OatA is important for the future development of effective inhibitors which may serve as antivirulence agents.

## Introduction

Multi-drug resistance amongst important human pathogens, such as methicillin-resistant *Staphylococcus aureus* (MRSA), vancomycin-resistant *Enterococcus* (VRE) and drug-resistant *Streptococcus pneumoniae*, continues to challenge clinicians and threaten the lives of infected patients to the extent that the United Nations recently endorsed a “Global Action Plan on antimicrobial resistance” (http://www.who.int/antimicrobial-resistance/publications/global-action-plan/en/). With the pipeline of traditional antibiotics all but dried up, alternative strategies are now being considered [1]. One novel approach to address future antimicrobial therapy is to exploit a well-established antimicrobial target in a new way that works synergistically with the natural host defenses, while minimizing deleterious effects on the beneficial community of commensal bacteria. An example of this involves sensitizing the cell walls of bacterial pathogens to attack by either host immune systems or endogenous lysins (autolysins) through the inhibition of a specific metabolic target enzyme.

**Fig 1:**
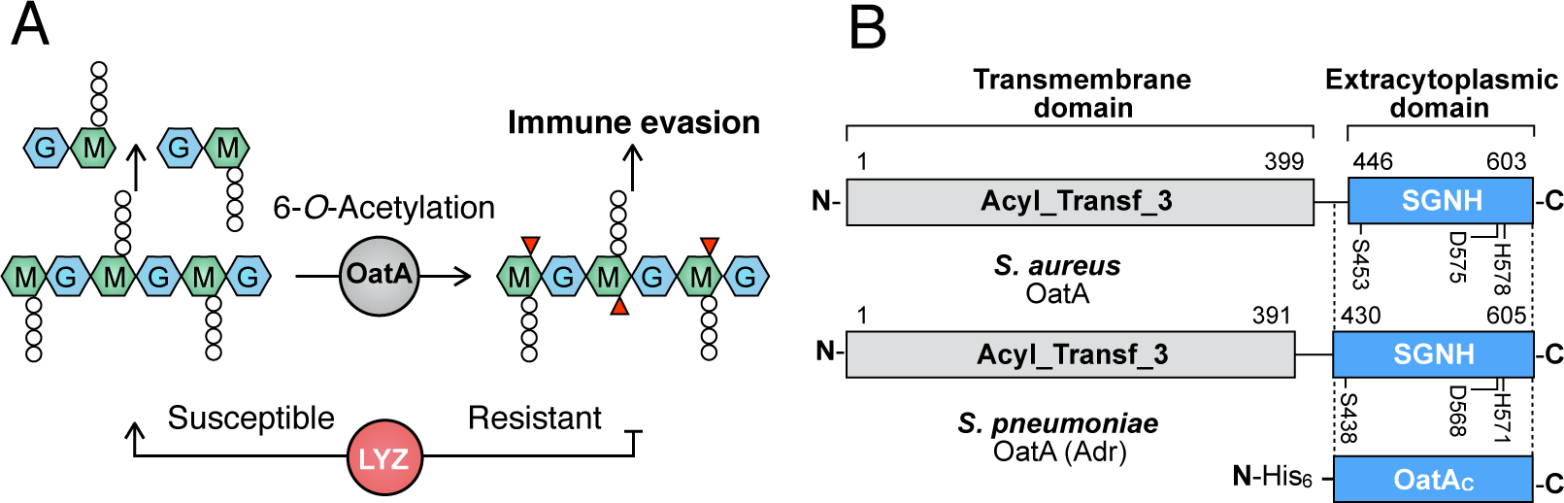
Activity and domain structure of OatA. **A**. PG is comprised of alternating GlcNAc (G) and MurNAc (M) residues with stem peptides (small circles). The lysozymes of innate immunity systems (LYZ) hydrolyze the linkage between M and G residues which results in cell rupture and death. OatA O-acetylates the C-6 hydroxyl group of MurNAc residues (red triangles) in PG of pathogenic Gram-positive bacteria which sterically inhibits the action of the lysozymes, thereby conferring resistance to this first line of the innate immune response. **B**. Domain organization of OatA. This bimodular protein is comprised of two domains, a predicted N-terminal Acyl_transferase_3 (Pfam PF01757) transmembrane domain and a C-terminal SGNH/GDSL extracytoplasmic domain. The genes encoding OatA from *S*. *aureus* and *S*. *pneumoniae* were engineered to produce the 25 kDa C-terminal SGNH/GDSL domains (OatA_C_) as shown.

The peptidoglycan (PG) sacculus is a key component of bacterial cell walls. PG encloses the cytoplasmic membrane to counter the turgor pressure of the cytoplasm thereby maintaining cell viability. Being both essential and unique to bacteria, PG is a prime target for the innate immune system, specifically through the production and release of lysozymes [2]. These enzymes hydrolyze the β-(1→4) linkage between the repeating *N-*acetylmuramoyl (MurNAc) and *N-*acetylglucosaminyl (GlcNAc) residues that form the glycan chains of PG (**Fig 1A**) thereby leading to rapid cell rupture and death. In the early stages of an infection, released PG fragments circulate in the host and serve as a critical activator of the immune system [3]. To defend against this host innate immune response, many pathogenic bacteria chemically modify their PG through O-acetylation.

The O-acetylation of PG occurs at the C-6 hydroxyl group of MurNAc residues and thereby sterically inhibits the productive binding of lysozyme [4,5] in a concentration dependent manner (reviewed in [6–9]). This PG modification exists in many Gram-positive and Gram-negative bacteria, but it appears to be particularly prevalent in pathogenic species. For example, only pathogenic species of *Staphylococcus*, including *S*. *aureus*, possess O-acetylated PG and each is highly resistant to lysozyme. On the other hand, non-pathogenic species lack this modification and they are lysozyme sensitive [10]. The extent of PG O-acetylation varies with species and strain, and typically ranges between 20% and 70% [6–9]. The age of a bacterial culture also appears to influence PG O-acetylation. For example, increases in O-acetylation of 10–40% were observed with cultures of *Enterococcus faecalis* entering stationary phase and a further 10–16% when cells become viable but non-culturable [11]. The increased susceptibility of PG with decreased levels of O-acetylation to host lysozyme has been demonstrated to correlate directly with the decrease in pathogenicity of, *e*.g., *S*. *aureus* [10,12,13], *Streptococcus suis* [14], *S*. *pneumoniae* [15], *Streptococcus iniae* [16], *E*. *faecalis* [17,18], *Listeria monocytogenes* [19–21], *Helicobacter pylori* [22], and *Neisseria meningitidis* [23]. With each of these pathogens, the enzyme directly responsible for PG O-acetylation and/or its regulator(s) was identified as a critical virulence factor.

The enzyme catalyzing the O-acetylation of PG in Gram-positive bacteria was first identified in *S*. *aureus* over ten years ago as *O*-acetyltransferase (Oat) A [12]. Homologs of OatA from several other Gram-positive bacteria have since been characterized genetically and phenotypically, including those from: clinical isolates of *S*. *pneumoniae* [15,24], *Bacillus cereus* [25], *E*. *faecalis* [26], *Lactobacillus plantarum* [27] and *L*. *monocytogenes* [20]. In addition to providing increased resistance to lysozyme [10,12–22], OatA activity is known to attenuate resistance to ß-lactam antibiotics [15], control endogenous autolytic activity [11,21,26,27], and control cell septation [27]. Despite this recognition and its importance as a major virulence factor [10–23], little is known about OatA at the molecular level. It is predicted to be bimodular, being comprised of an N-terminal integral membrane domain linked to a C-terminal extracytoplasmic domain [28]. Based on analogy to the two component PG O-acetylation system in Gram-negative bacteria, which involves an integral membrane acetyltransporter (PatA) and a cytoplasmic *O-*acetyltransferase (PatB) [6,8,29], the surface-exposed C-terminal region of OatA is postulated to function as the *O*-acetyltransferase. There is minimal sequence similarity between the C-terminal domain of OatA and the well-characterized PatB [29–32] (*eg*. 15.4% identity and 18.3% similarity between *N*. *gonorrhoeae* PatB and the C-terminal domain of *S*. *aureus* OatA). Moreover, no biochemical analysis of OatA has been reported. A lysine rich region in the C-terminal domain was postulated to contain the active site [10] but closer analysis of its predicted amino acid sequence suggests that it has the fold of SGNH/GDSL hydrolases with a signature catalytic triad of Asp, His and Ser residues [6,28]. However, to date the crystal structure of peptidoglycan *O*-acetyltransferase (*i*.*e*., PatB, OatA) from any bacterium remains unknown.

## Results

### The extracytoplasmic domain of OatA (OatA_C_) functions as an *O-*acetyltransferase

**Fig 2:**
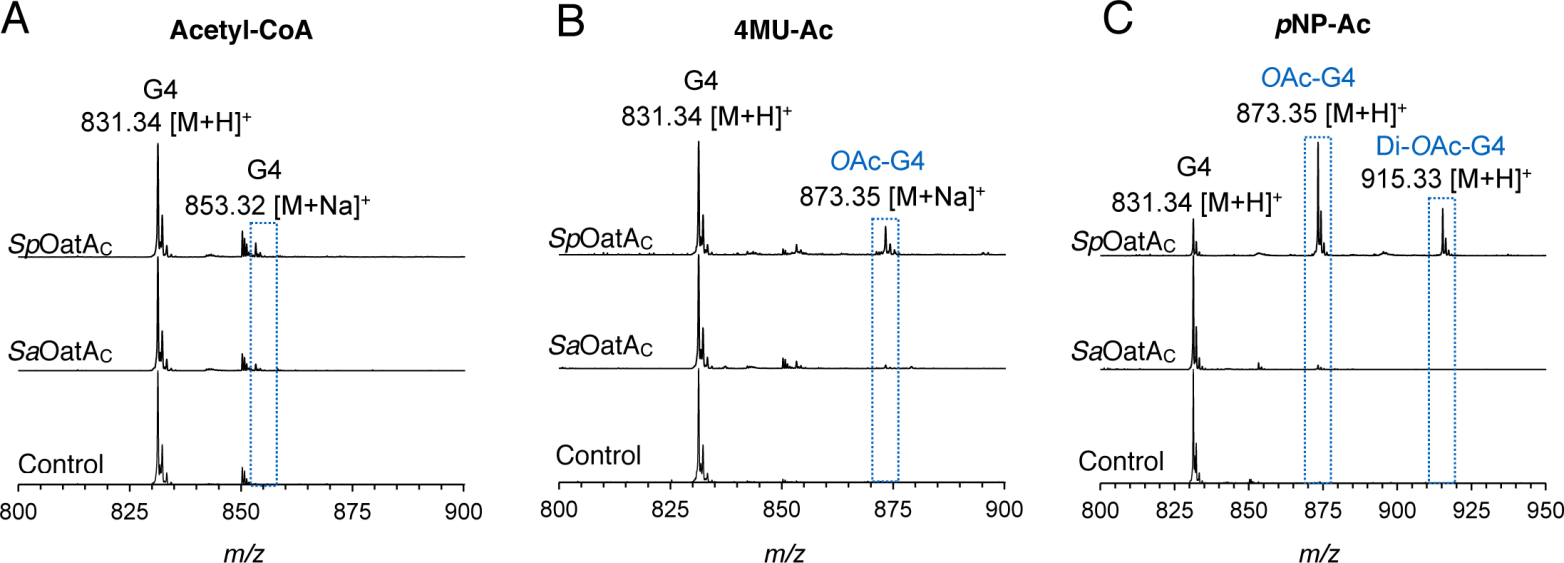
*Sp*OatA_C_ and *Sa*OatA_C_-catalyzed *O*-acetyltransferase reactions. ESI-MS analysis of reaction products of 2 mM chitotetraose (G_4_) in 50 mM sodium phosphate buffer pH 6.5 incubated at 37 ^o^C for 1 h in the absence (control) and presence of enzymes (5 μM, final concentration) with 1 mM concentrations of **A,** acetyl-CoA; **B,** 4MU-Ac; or **C,** *p*NP-Ac as potential donor acetyl substrates.

To provide soluble forms of OatA homologs suitable for *in vitro* studies, the recombinant C-termini of the proteins from *S*. *aureus* (S*a*OatA_C_; residues 435–603) and *S*. *pneumoniae* (*Sp*OatA_C_; residues 423–605) (**Fig 1B**) were produced and purified to apparent homogeneity, as judged by SDS PAGE (**S1 Fig**). The amino acid sequences of these homologs share 28.5% identity and 54.1% similarity. We investigated their catalytic activity using our previously described qualitative assay for PG *O*-acetyltransferases [30,31] with pseudosubstrates *p*-nitrophenylacetate (*p*NP-Ac), 4-methylumbelliferylacetate (4MU-Ac) or acetyl-CoA as acetyl-donors, and chitotetraose (GlcNAc_4_) as the acetyl acceptor. We used electrospray ionization-mass spectrometry (ESI-MS) analysis of reaction products to identify a single predominant O-acetylated chitotetraose product ion (*m*/*z* = 873.35 [M+H]^+^) produced by both enzymes only when *p*NP-Ac or 4MU-Ac were used (**Fig 2**), suggesting that acetyl-CoA is not a suitable donor. The respective reaction products were analyzed further by MS/MS which showed that both enzymes modified the terminal non-reducing GlcNAc residue of chitotetraose (**S2 Fig**). We observed a second O-acetylation by *Sp*OatA_C_ when using *p*NP-Ac as acetyl donor that occurred on one of the two internal GlcNAc residues which could be discerned by MS/MS. These data demonstrated that the extracytoplasmic C-terminal domains of OatA homologs function as *O*-acetyltransferases *in vitro* and that the activities are coupled to the turnover of the pseudosubstrates *p*NP-Ac or 4MU-Ac.

**Fig 3:**
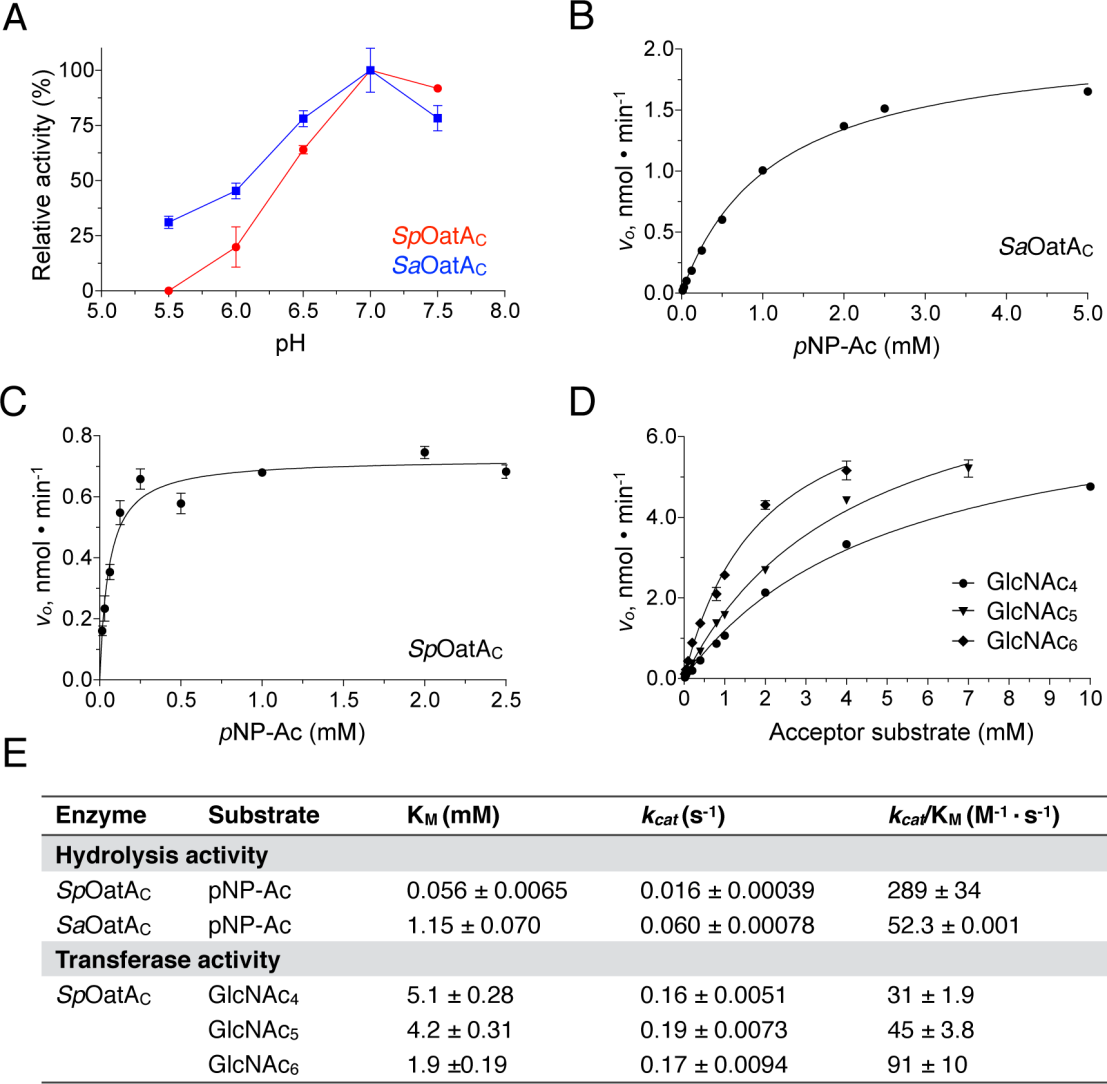
Kinetic analysis of *Sp*OatA_C_ and *Sa*OatA_C_-catalyzed *O*-acetyltransferase reactions. **A**. pH dependence of the esterase activity catalyzed by *Sp*OatA_C_ (red) and *Sa*OatA_C_ (blue). The specific activities of the enzymes were determined in 20 mM sodium citrate-phosphate-borate buffer at the pH values indicated at 25 ^o^C. **B, C**. Determination of the steady-state parameters for the esterase activities of *Sa*OatA_C_ and *Sp*OatA_C_, respectively. Initial velocities of *p*NP-Ac hydrolysis were determined for the respective enzymes (5 μM) in 50 mM sodium phosphate buffer pH 6.5 containing 5% (*v/v*) ethanol at 25 ^o^C. **D**. Determination of the steady state-parameters for the *O*-acetyltransferase activity of *Sp*OatA_C_ on chitooligosaccharides. The initial velocities of acetyl transfer to the chito-oligosaccharides at the concentrations indicated were determined using 5 μM enzyme in 50 mM sodium phosphate buffer pH 6.5 at 25 ^o^C with *p*NP-Ac fixed at 2 mM. **E**. Michaelis-Menten parameters determined for *Sp*OatA_C_ and *Sa*OatA_C_ from the experiments presented in panels **B**, **C** and **D**. All of the enzymatic experiments were performed in triplicate, with the s.e. noted.

In the absence of acceptor substrate, *Sp*OatA_C_ and *Sa*OatA_C_ exhibited weak hydrolase activity toward *p*NP-Ac and 4MU-Ac. We used this esterase activity to determine that the pH-activity optimum for both enzymes was 6.8 (**Fig 3A**). Steady-state kinetic analyses provided similar Michaelis-Menten parameters for hydrolysis of *p*NP-Ac by each enzyme with *Sp*OatA_C_ being approximately 3-fold more efficient than *Sa*OatA_C_, as reflected by *k*_cat_/*K*_M_ values (**Fig 3B, 3C and 3E**). However, as esterases the two enzymes were 252 and 45-fold less efficient, respectively, than authentic *O*-acetyl-PG esterase (Ape) from *N*. *gonorrhoeae* under similar conditions [33].

### OatA_C_ has specificity for glycan chains

Accounting for the slower rates of hydrolysis in the absence of acceptors, we could determine the kinetics of *O*-acetyl transfer to various acceptors [31] (**S3 Fig**). We used this assay to investigate the specificity of the enzymes for acceptor chain lengths. Initial experiments involved chito-oligosaccharides with degrees of polymerization (DP) between 2 and 6 as acceptors. With *Sp*OatA_C_, its specific activity did not increase above the rate of hydrolysis when incubated in the presence of chito-oligosaccharides with a DP ≤ 3. However, the inclusion of oligomers with a DP ≥ 4 significantly enhanced the rates of donor acetyl turnover. Confirmation that these GlcNAc oligomers served as acceptors was obtained by MS analysis (**S4 Fig**). We attempted to determine the steady-state kinetics of the *O-*acetyltransferase activity with these chito-oligosaccharides but their limited solubility precluded our ability to provide saturating concentrations (**Fig 3D**). Consequently, the kinetic parameters presented in **Fig 3E** were obtained by extrapolation of the Michaelis-Menten regression curves. Despite this limitation, the data suggested that *Sp*OatA_C_ has specificity for longer acceptor substrates as increased *k*_cat_/*K*_m_ values were obtained with increasing acceptor DP. Furthermore, comparison of *k*_cat_ values indicated that *Sp*OatA_C_ functions at least an order of magnitude faster as an *O-*acetyltrasferase than as an esterase. In contrast, reactions catalyzed by *Sa*OatA_C_ were not significantly influenced by any of the chito-oligosaccharide acceptors tested (**S3B Fig**) suggesting this homolog may have a higher specificity for more complex acceptor substrates.

### Preparation of a muroglycan-based homopolymer substrate

In an attempt to confirm that both *Sa*OatA_C_ and *Sp*OatA_C_ function as PG *O-*acetyltransferases, we tested their activity using our MS-based assay developed previously for the study of PatB [30,31]. This assay uses a pool of soluble muroglycans prepared by the limited mutanolysin digestion of purified PG as acceptor substrate. To our surprise, we could not detect any O-acetylated products in reaction mixtures following incubation of either OatA_C_ with this heterogenous pool of muroglycans and either *p*NP-Ac or 4MU-Ac as acetyl donor. With the failure of this assay, we wondered if OatA has a more rigid specificity for muroglycans compared to PatB with respect to composition and/or DP. Unfortunately, it is technically challenging to prepare defined muroglycan substrates from natural sources of PG in sufficient quantity for study given the inherent heterogeneity of stem peptide composition, extent of cross-linking, and post-synthetic modifications. To circumvent this, we prepared a novel substrate *in vitro* that is a linear homopolymer of the natural precursor for PG biosynthesis, Lipid II. Recombinant penicillin-binding protein (PBP) 2a from *S*. *pneumoniae* was produced [34] and used to polymerize Lipid II under varying buffer conditions. The resulting linear homopolymers (muroglycan-5P) consisted of repeating units of GlcNAc-MurNAc-l-Ala-d-Glu-l-Lys-d-Ala-d-Ala (GM-pentapeptide) linked to an undecaprenyl pyrophosphate (UndP) through C1 of the reducing MurNAc residue (**S5 Fig**). Muroglycan-5P remained uncrosslinked because the transpeptidase domain of *S*. *pneumoniae* PBP2a is only active on stem pentapeptides containing amidated d-Glu residues (iso-d-Gln); this amidation is conferred *in vivo* by Lipid II amidotransferase [34].

We found that the PBP2a-catalyzed polymerization of Lipid II could be controlled by detergent concentration (**S6 Fig**). With 0.04% (*v/v*) Triton X-100, a pool of muroglycans-5P enriched with DP 2–10 was generated. Whereas these muroglycans presented a suitable potential substrate for subsequent O-acetylation reactions, the presence of the residual UndP moiety at their reducing ends interfered with their MS analysis. Consequently, we digested samples with a muramidase (mutanolysin) prior to MS.

### *Sp*OatA_C_ and *Sa*OatA_C_ have different specificities for muroglycans

**Fig 4:**
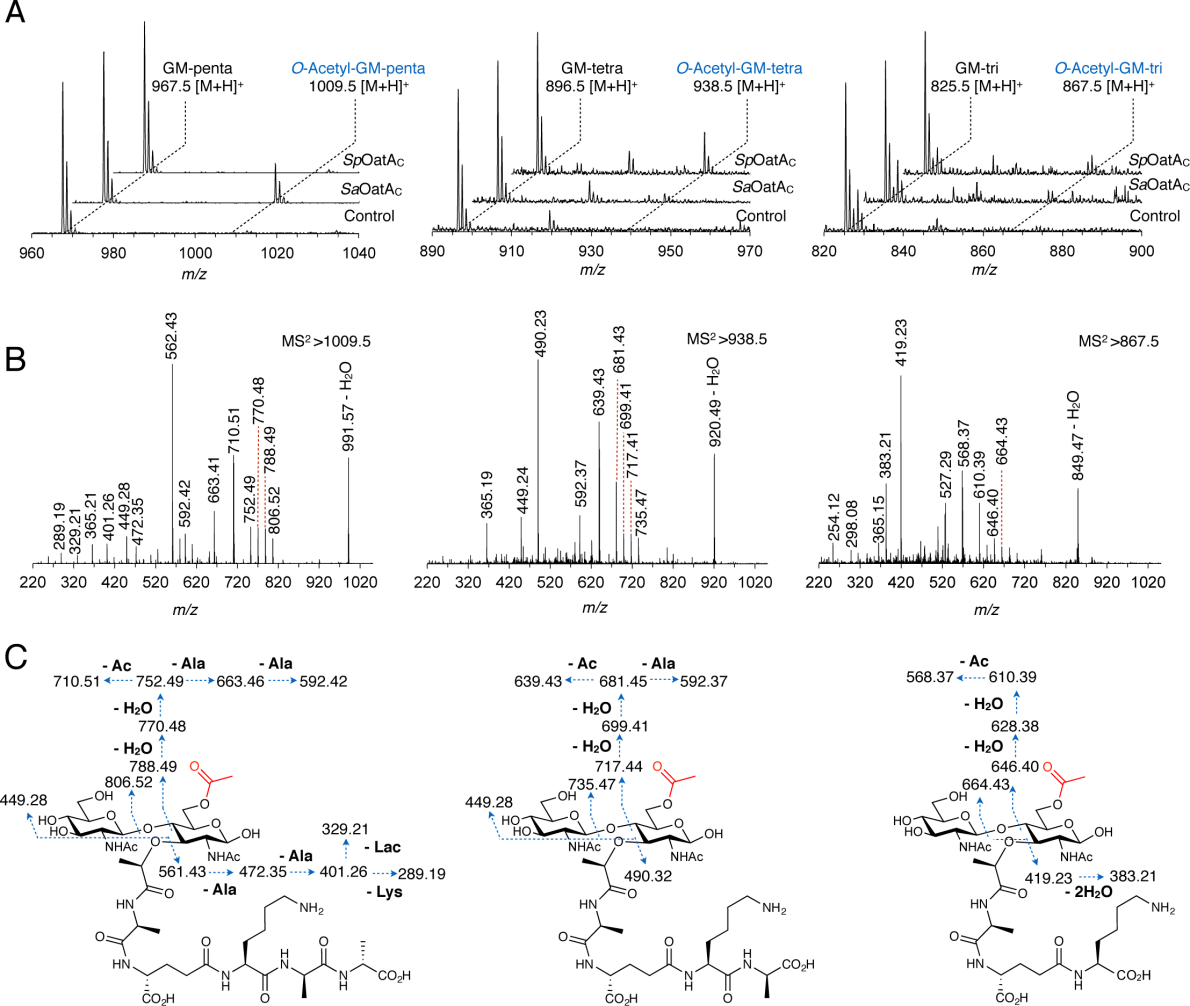
Stem peptide specificity of *Sp*OatA_C_ and *Sa*OatA_C_. **A**. Stacked and offset ESI-mass spectra of mutanolysin-treated products from reactions of 10 μg·mL^-1^ of (left to right) muroglyan-5P, muroglycan-4P, and muroglycan-3P in 50 mM sodium phosphate buffer pH 6.5 incubated with 0.5 mM *p*NP-Ac in the absence (control) and presence of the respective enzyme (10 μM). The major O-acetylated products are labeled in blue which are 42.01 m/z units larger than the respective unmodified PG monomer. **B**. MS/MS analysis of the major product ions identified in the respective panels above and **C**, interpretation of the corresponding fragment ions.

We used muroglycan-5P to characterize the substrate specificity of the two OatA_C_ homologs. MS analysis of a sample of muroglycan-5P (DP 4–10) incubated with *Sa*OatA_C_ in the presence of *p*NP-Ac followed by mutanolysin digestion revealed a new prominent ion (*m*/*z* = 1009.45 [M+H]^+^) 42.01 mass units larger than GM-pentapeptide (m/z = 967.44 [M+H]^+^) which corresponds to an O-acetylated product (**Fig 4A**). MS/MS analysis verified this O-acetylation and that it occurred only on MurNAc residues (**Fig 4B and 4C**). The O-acetylated product was not observed in reactions with monomeric GM-pentapeptide that had been generated *in situ* by mutanolysin digestion prior to incubation with *Sa*OatA_C_. Similarly, commercially available GM-dipeptide did not serve as an acceptor substrate for the enzyme. These data confirmed that *Sa*OatA_C_ functions as a PG *O-*acetyltransferase, and that it has specificity for the MurNAc residues within PG glycan chains.

Given the lack of apparent activity toward the heterogeneous mix of sacculus-derived muroglycans (as described above), we wondered if the substrate specificity of *Sa*OatA_C_ extended to the stem peptide of PG strands. To investigate this, samples of the muroglycan-5P were incubated with recombinant d,d-carboxypeptidase DacA (PBP3) [35] to provide uniformly tailored muroglycans of GM-tetrapeptide repeats (muroglycan-4P). Samples of these muroglycans-4P were then treated with l,d-carboxypeptidase DacB (LdcB) [35] to provide further trimmed muroglycans with GM-tripeptide repeats (muroglycan-3P). ESI-MS confirmed the production of the respective muroglycan pools. Interestingly, neither muroglycan-4P nor muroglycan-3P served as effective acceptors for *Sa*OatA_C_ as very little product was observed with each (**Fig 4A**). These results showed that this *O-*acetyltransferase has specificity for the pentapeptide stems on PG, a form of the muropeptide unit that would present in very low concentrations within mature PG.

Parallel assays with *Sp*OatA_C_ revealed that it too only O-acetylates the MurNAc residues of muroglycans, but its substrate specificity with respect to stem peptide composition was distinctly different. In contrast to *Sa*OatA_C_, *Sp*OatA_C_ was inactive against muroglycan-5P (**Fig 4A**). Instead, it had a strong preference for muroglycan-4P and, like *Sa*OatA_C_, was only weakly active on muroglycan-3P. Again, MS/MS analysis confirmed the specific O-acetylation of MurNAc residues (**Fig 4B and 4C**). Taken together, these experiments demonstrated that the stem peptide composition of PG glycan chains has a significant effect on substrate recognition by the extracytoplasmic domains of OatA.

### *Sp*OatA_C_ has an SGNH/GDSL hydrolase fold with a canonical catalytic triad

To gain insight into the mechanism of action of OatA_C_, we undertook structural analysis of the enzyme using X-ray crystallographic techniques. Although, both *Sa*OatA_C_ and *Sp*OatA_C_ were subjected to crystallization trails, *Sa*OatA_C_ proved recalcitrant to crystallization. *Sp*OatA_C_ crystallized in both the native and SeMet derivative forms and crystals diffracted to 1.12 Å and 1.8 Å resolution, respectively (**S1 Table**). We solved the structure using single-wavelength anomalous dispersion method and it was subsequently used as a search model for phasing the native high resolution diffraction data using molecular replacement method. The native enzyme was refined to *R*_work_/*R*_free_ values of 15.0/16.8% (**S1 Table**).

**Fig 5:**
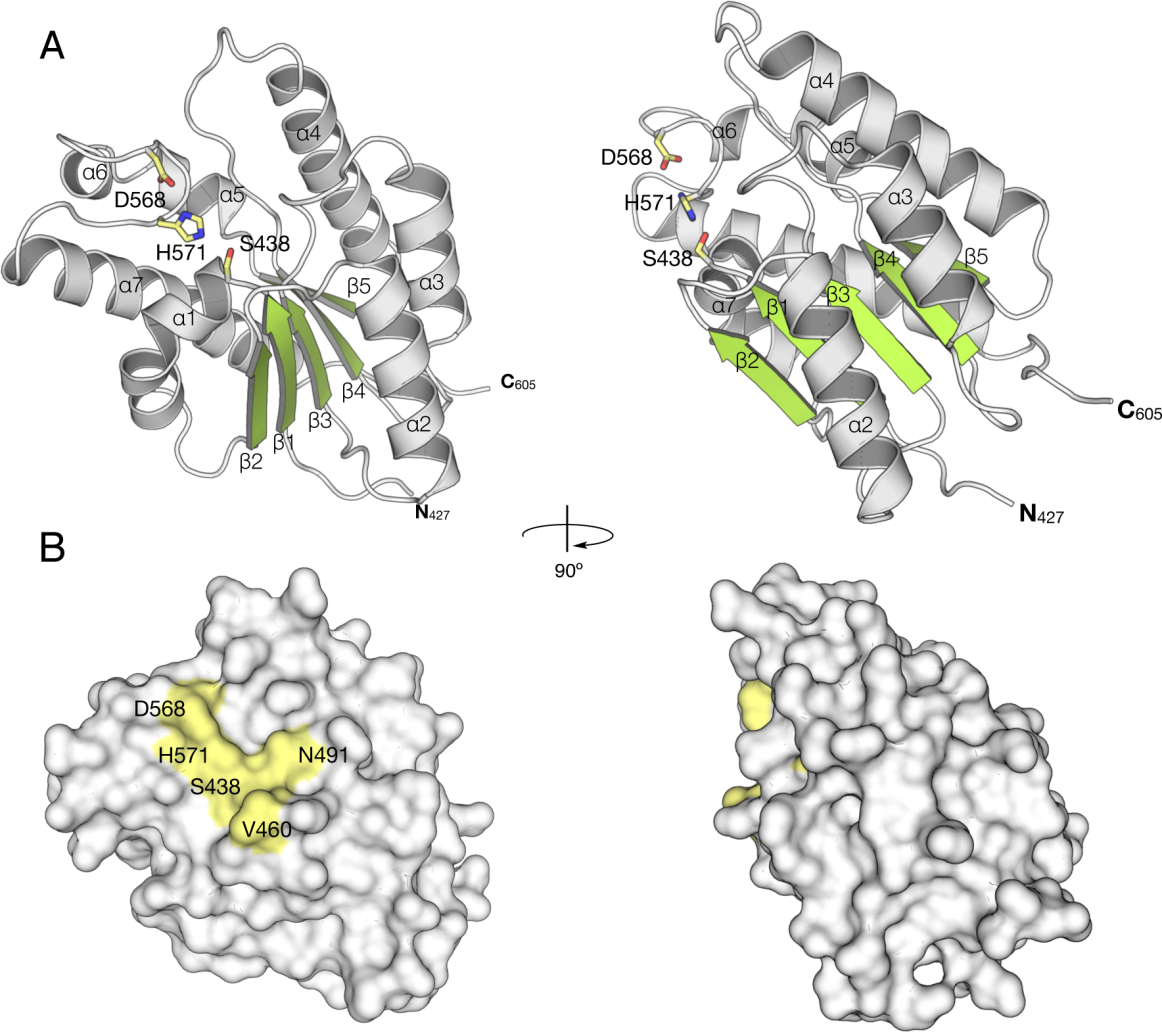
Structure of *Sp*OatA_C_. **A**. Cartoon representations identifying the seven α-helices (gray), five β-strands (green), and coils (white) of the atypical α/β hydrolase fold adopted by *Sp*OatA_C_. Also, shown in stick form are the amino acids comprising the active-site and oxyanion hole (PDB ID: 5UFY). **B**. Surface representations of *Sp*OatA_C_ depicting the putative catalytic triad and oxyanion hole residues within a shallow pocket of the enzyme.

The overall structure of *Sp*OatA_C_ adopts an atypical α/β hydrolase fold (**Fig 5A**), where the core parallel β-sheet contains five strands (β1 - β5) sandwiched between seven α-helices (α1–α7) forming a shallow and solvent exposed putative active site pocket (**Fig 5B**). A structural similarity search using the DALI server revealed that *Sp*OatA_C_ most closely resembles *Bos taurus* platelet-activating factor acetylhydrolase [36] (PDB ID: 1BWQ; RMSD 2.4 Å over 158 residues) and *Escherichia coli* thioesterase I/ protease I/ lysophospholipase L1 [37] (PDB ID: 1IVN; RMSD 2.9 Å over 156 residues), two members of the SGNH/GDSL hydrolase superfamily (cl01053). Notable similarities were also seen with the SGNH/GDSL hydrolase Ape from *N*. *meningitidis* [38] (PDB ID: 4K40; RMSD 2.9 Å over 155 residues) and rhamnogalacturonan acetylesterase from *Aspergillus aculeatus* [39] (PDB ID: 1DEO; RMSD 3.2 Å over 153 residues). Our identification of *Sp*OatA_C_ as a member of the SGNH/GDSL hydrolases is consistent with previous predictions regarding the structures of *L*. *plantarum* OatA [28] and *N*. *gonorrhoeae* PG *O-*acetyltransferase (PatB) [32]. Interestingly however, the DALI algorithm did not identify isoamyl acetate hydrolyzing esterase from *Saccharomyces cerevisiae* as a close homolog of *Sp*OatA_C_; this other member of the SGNH/GDSL hydrolases was used by the algorithm to predict the structure of *N*. *gonorrhoeae* PatB [32].

That *Sp*OatA_C_ adopts a true SGNH hydrolase fold distinguishes it from the SGNH hydrolase-like structures of the alginate *O*-acetyltransferases of *Pseudomonas aeruginosa* (*Pa*AlgX (PDB ID:); identity: 11%, Cα RMSD: 3.6 Å over 79 residues) and *Pseudomonas putida* (*Pp*AlgJ (PDB ID:); identity: 15%, RMSD: 3.1 Å over 92 residues), as well as *Bacillus cereus* secondary cell wall polysaccharide *O*-acetyltranferase (*Bc*PatB1 (PDB ID: 5V8E); identity: 11%, Cα RMSD: 3.6 Å over 79 residues). Although their folds are similar, topologically these latter enzymes are different from *Sp*OatA_C_ due to a circular permutation of their amino acid sequences, which characterizes them as part of the AlgX_N-like superfamily (*Pa*AlgX and *Pp*AlgJ; cl16774) and the DHHW superfamily (*Bc*PatB1; cl25368), respectively.

**Fig 6:**
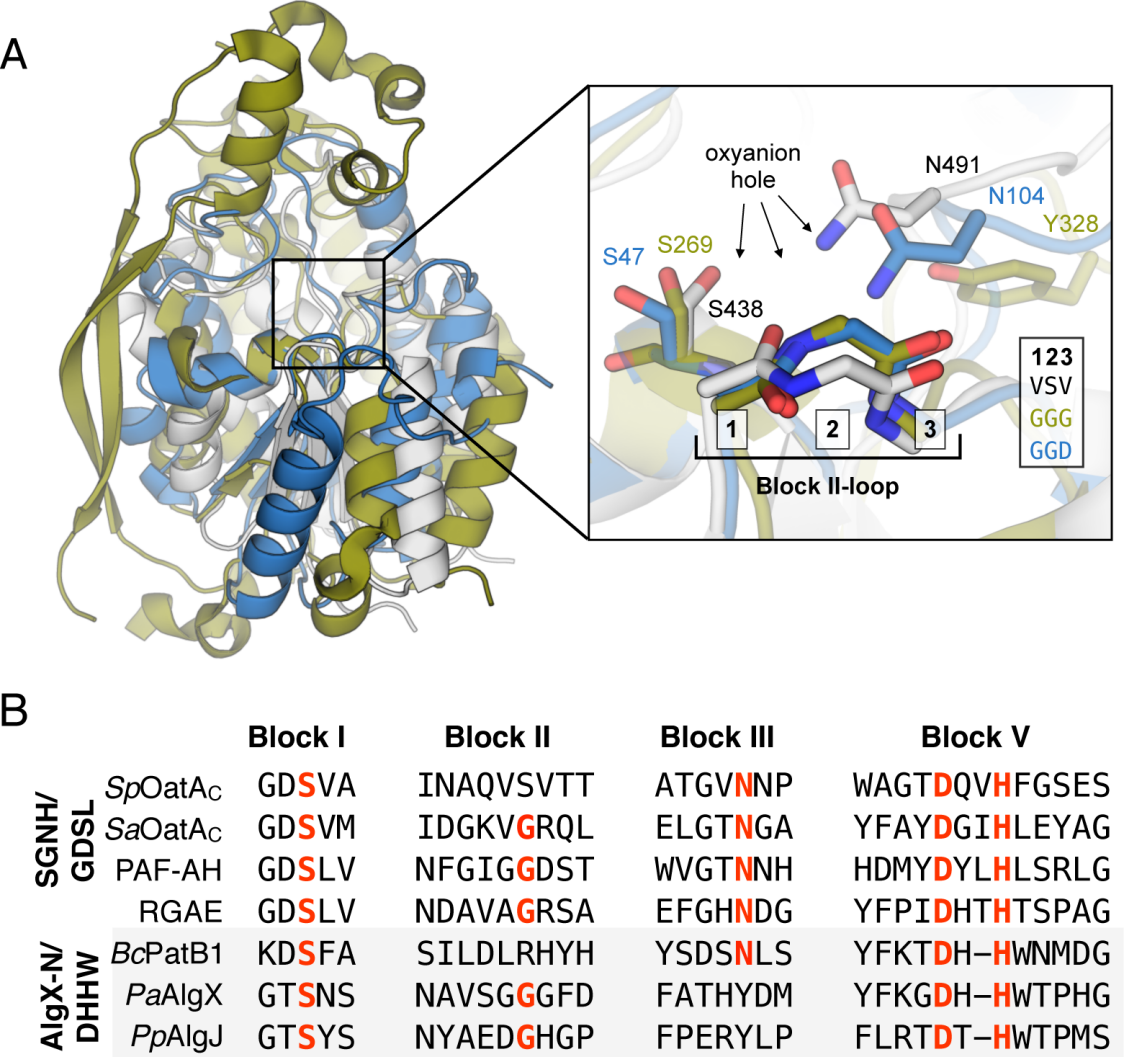
Structural comparison of *Sp*OatA_C_ with representative members of the SGNH/GDSL and AlgX-N/DHHW families of enzymes. **A**. The cartoon representation of *Sp*OatA_C_ (gray) is superposed with *Bos taurus* platelet-activating factor acetylhydrolase (PAF-AH) (blue) and the N-terminal catalytic domain of *P*. *aeruginosa* AlgX (green). Right inset: Cartoons depicting the respective peptide backbones of the Block II-loop in the three enzymes. **B**. Sequence alignments of residues comprising the signature sequence Blocks of the SGNH/GDSL and AlgX-N/DHHW families of enzymes. Red lettering denotes invariant residues in the respective families.

On the whole, the true SGNH hydrolases (esterase or transferase) share low sequence similarity, but they are characterized by four consensus sequence Blocks (I, II, III, V) [40] (**Fig 6B**). Almost all members of this family contain a conserved catalytic triad formed by a Ser nucleophile from Block I and conserved His and Asp residues from Block V. The homologous residues in *Sp*OatA_C_ are indeed aligned appropriately to serve as a catalytic triad (**Fig 5A**). Ser438 is engaged in an H-bond network with His571 and Asp568 and the triad is positioned in the center of the putative active-site cleft of the enzyme (**Fig 5B**). To verify their role as catalytic residues, we performed site-directed mutagenesis on the *oatA*_*C*_ genes and kinetically characterized the recombinant variants. The S438A and H571A *Sp*OatA_C_ variants were devoid of detectable *O-*acetyltransferase activity while replacement of Asp568 with Asn resulted in minimal catalytic activity (**Table** 1). Our replacement of the equivalent residues in *Sa*OatA_C_ produced similar results. Unequivocal identification of Ser438 in *Sp*OatA_C_ as the catalytic nucleophile was made using the mechanism-based, irreversible inhibitor of Ser esterases methanesulfonyl fluoride (MSF) [41]. *Sp*OatA_C_ treated with this reagent lacked detectable catalytic activity and X-ray crystallographic analysis of a native crystal soaked with MSF revealed the formation of a covalent adduct to Ser438 (described further below).

**Table 1:** Specific activities of *Sp*OatA_C_ and *Sa*OatA_C_ variants.

Enzyme	Specific activity (nmol⋅min^-1^⋅mg^-1^)^1^			
	Hydrolysis^2^		Transfer^3^	
*Sp*OatA_C_				
Wild-type	14.6 ± 2.4	(100%)	163 ± 3.3	(100%)
D568N	1.9 ± 0.3	(13%)	n.d.	(0%)
H571A	n.d.	(0%)	n.d.	(0%)
S438A	n.d.	(0%)	n.d.	(0%)
N491A	6.1 ± 0.15	(41.8%)	n.d.	(0%)
V460G	21.6 ± 0.82	(148%)	16.1 ± 1.1	(9.9%)
V460A	17.6 ± 0.97	(121%)	34.4 ± 3.3	(21.1%)
V460I	21.6 ± 0.76	(148%)	291 ± 8.8	(178%)
*Sa*OatA_C_				
Wild-type	29.2 ± 0.3	(100%)	–	–
D575A	3.1 ± 0.1	(10.6%)	–	–
H578A	n.d.	(0%)	–	–
S453A	n.d.	(0%)	–	–

^1^Presented as means ± standard error (n = 3).

^2^ Reactions conducted in 50 mM sodium phosphate buffer, pH 6.5 at 25°C with 1 mM *p*NP-Ac.

^3^Same conditions as above but including 5 mM chitopentaose as acceptor.

n.d., not detected; (—), not determined.

### *Sp*OatA_C_ possesses an atypical two-residue oxyanion hole

A characteristic of the SGNH/GDSL hydrolases is the presence of an oxyanion hole comprised of the signature residues as H-bond donors: (i) the backbone NH of the Ser nucleophile; (ii) the backbone NH of Gly from consensus Block II, and (iii) the side chain amide of Asn from Block III (**Fig 6B**). The residues of Block II form a type-II β-turn, which positions the backbone NH of the conserved Gly toward the active site so that it, together with the Ser and Asn residues, can serve its role in the oxyanion hole as an H-bond donor to stabilizes the oxyanion of the tetrahedral intermediate. The AlgX-like enzymes possess a similar Block II geometry, but lack the Asn in Block III that functions as the third H-bond donor (**Fig 6A and 6B**).

**Fig 7:**
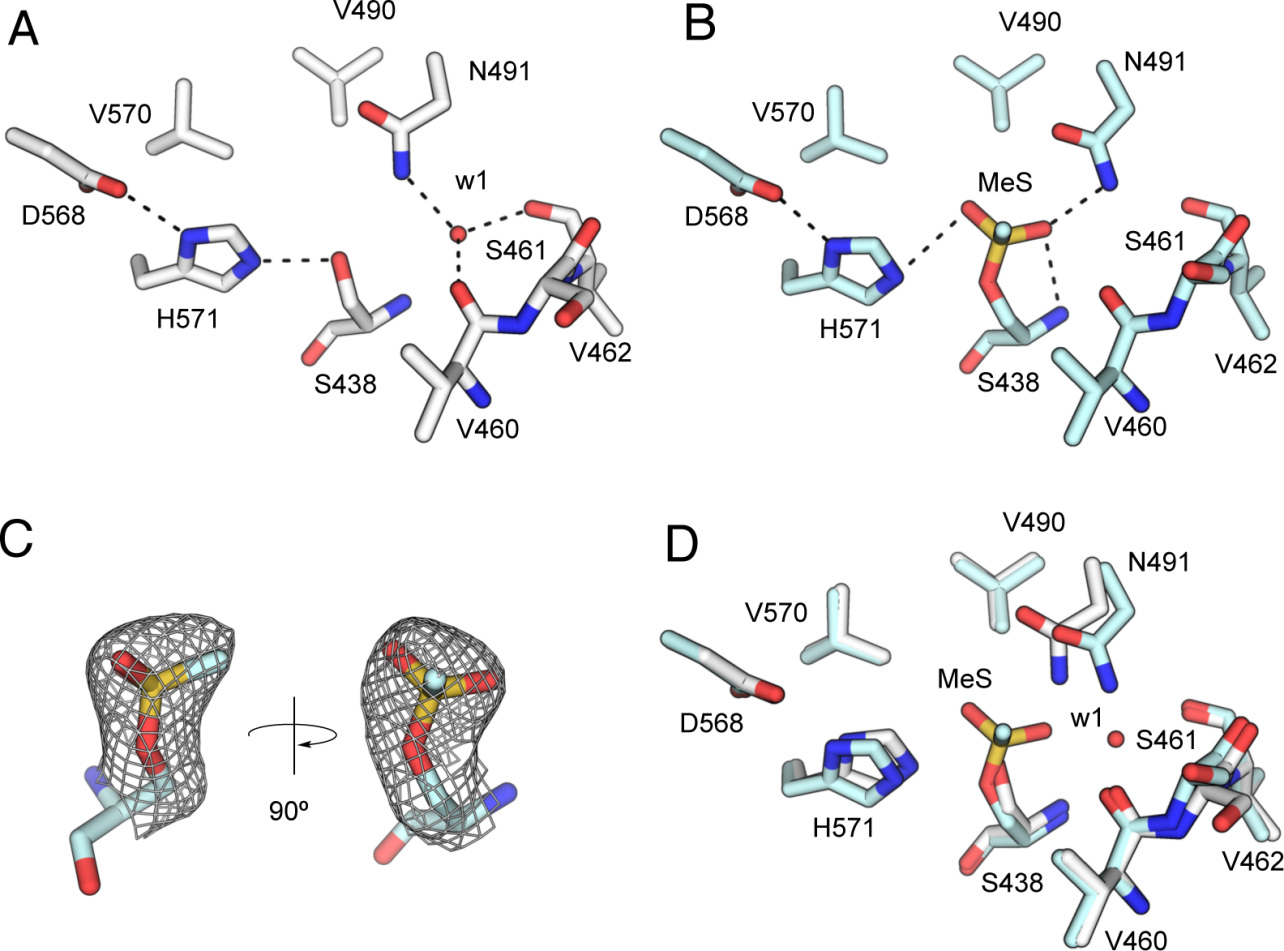
Active site structure of *Sp*OatA_C_. The H-bonding network of catalytic and oxyanion hole residues in **A**, resting *Sp*OatA_C_ and **B**, *Sp*OatA_C_ in complex with MeS (*Sp*OatA_C_-MeS). The water molecule w1 and the potential inter-residue interactions are depicted as a red sphere and black dashed lines, respectively. **C**. The *2F*_*o*_*-F*_*c*_ electron density map of the MeS-Ser438 adduct contoured at 1.0 σ. **D**. Superposition of the *Sp*OatA_C_ and *Sp*OatA_C_-MeS active sites.

The oxyanion hole structure in *Sp*OatA_C_ is distinct from other SGNH hydrolases. Whereas *Sp*OatA_C_ possesses the Ser nucleophile and an Asn in Block III (Asn491 in *Sp*OatA_C_), the conserved Gly of Block II is replaced with a Ser (Ser461) (**Fig 6B**). Moreover, rather than forming a type-II β-turn, the residues of Block II in *Sp*OatA_C_ adopt a type-I β-turn resulting in 180° rotation of the peptide bond (**Fig 6A**). This orients the backbone carbonyl oxygen of Val460 (rather than backbone NH of Ser461) toward the active center where, together with the carbonyl oxygen of Val462, it coordinates a water molecule (w1) (**Fig 7A**). This water serves as an H-bond acceptor for Nδ2 of Asn491and thereby stabilizes the resting conformation of this oxyanion hole residue.

Another distinguishing feature of the oxyanion hole in *Sp*OatA_C_ is the positioning of its two component residues, Ser438 and Asn491 (**Fig 7A**). The Nδ2 of Asn491 is positioned 3.4 Å from Ser438 Oδ, a distance significantly closer than the average 5.4 Å reported for the equivalent distance between the homologous residues in SGNH/GDSL hydrolases. We confirmed the importance of Asn491 in catalysis by generating an N491A variant of *Sp*OatA_C_ which lacked detectable transferase activity under the conditions tested **(Table 1**). Unexpectedly, however, the variant retained 45% activity as a hydrolase.

### Asn491 is positioned differently in resting and active conformational states

The methylsulfonyl (MeS) adduct resulting from the inactivation of *Sp*OatA_C_ by MSF (described above) represents a transition-state analogue mimicking the attack of water on the acetyl-enzyme intermediate during hydrolysis. To confirm the identification of the catalytic nucleophile and gain further insight into the enzyme’s mechanism of action, we determined the structure of MSF-inactivated *Sp*OatA_C_ (*Sp*OatA_C_-MeS) at 2.1 Å resolution (**S1 Table**) following modification of the native enzyme *in cyrstallo* with MSF. The MeS group is seen to form a covalent bond to the Oδ of Ser438 in a tetrahedral configuration (**Fig 7B**) and it is well defined in the electron density map (**Fig 7C, S7 Fig**). The overall structures of *Sp*OatA_C_ and *Sp*OatA_C_-MeS are very similar (Cα RMSD: 0.327 Å over 178 residues), but several side chain displacements were observed in the active site on MeS binding. The most significant structural change involved the side chain of Asn491 (**Fig 7D**). The presence of the MeS adduct opens the active site, shifting the Nδ2 of Asn491 2 Å from its initial position away from Ser438 and displacing the water molecule w1 (**Fig 7A, 7B and 7D**). Other minor displacements occur in Ser438, Ser461, and His571 as a consequence of the steric effects imposed by the bound MeS.

The methyl group of the MeS adduct appears to face the solvent adjacent to Val460, and the sulfonyl O2 (the structural mimic of an attacking water for hydrolytic activity; labeled O11 in the coordinate file) forms a weak H-bond (3.3 Å) to the imidazolium group of His571 while protruding into a hydrophobic pocket formed by Val490 and Val570 (**Fig 7B**). Additionally, two H-bonds are made with the O1 of MeS (the structural mimic of the carbonyl O of a bound acetyl group; labeled O12 in the coordinate file), one involving the backbone NH of Ser438 and the other with Nδ2 of Asn491. These interactions are consistent with our earlier identification of Ser438 and Asn491 as comprising the oxyanion hole.

### Conserved Val460 promotes transferase activity

Our alignment of Block II sequences of OatA homologs revealed the existence of an invariant Val/Ile residue at position five (Val460 and Val475 of *Sp*OatA and *Sa*OatA, respectively) (**S8 Fig**) that is not conserved in SGNH-GDSL esterase members. We probed the importance of Val at this position in *Sp*OatA_C_ by its site-specific replacement with the amino acids located in the same position in the esterases, platelet-activating factor acetyl hydrolase (Gly) and rhamnogalacturonan acetyl esterase (Ala) (**Fig 6B**). The specific activity of both the V460G and V460A *Sp*OatA_C_ variants as esterases was slightly increased compared to the wild-type enzyme while a 10- and 5-fold reduction in transferase activity was observed, respectively (**Table 1**). Interestingly, replacement of Val460 with Ile, the only other residue found in this position in some OatA homologs, resulted in an increase in both activities, but the enhancement of *O*-acetyltransferase activity was significantly greater. Taken together, these data suggest that Val460 contributes to the effective binding of carbohydrate acceptor substrates.

## Discussion

One of the most common structural variations of PG produced by Gram-positive pathogens to protect them from lysis by innate immunity systems is the O-acetylation of MurNAc [6,7,42,43]. This modification to PG was first discovered almost 60 years ago [44] but the molecular details of the O-acetylation pathway remained unknown until now. In the current study, we show experimentally for the first time that the extracytoplasmic domains of OatA homologs from two important human pathogens function catalytically as *O*-acetyltransferases with specificity for both: i) the C6 hydroxyl group of MurNAc residues within muroglycan chains, and ii) the specific length of associated stem peptides. Additionally, our elucidation of the *Sp*OatA_C_ crystal structure has identified structural elements that are required for its catalytic mechanism.

**Fig 8:**
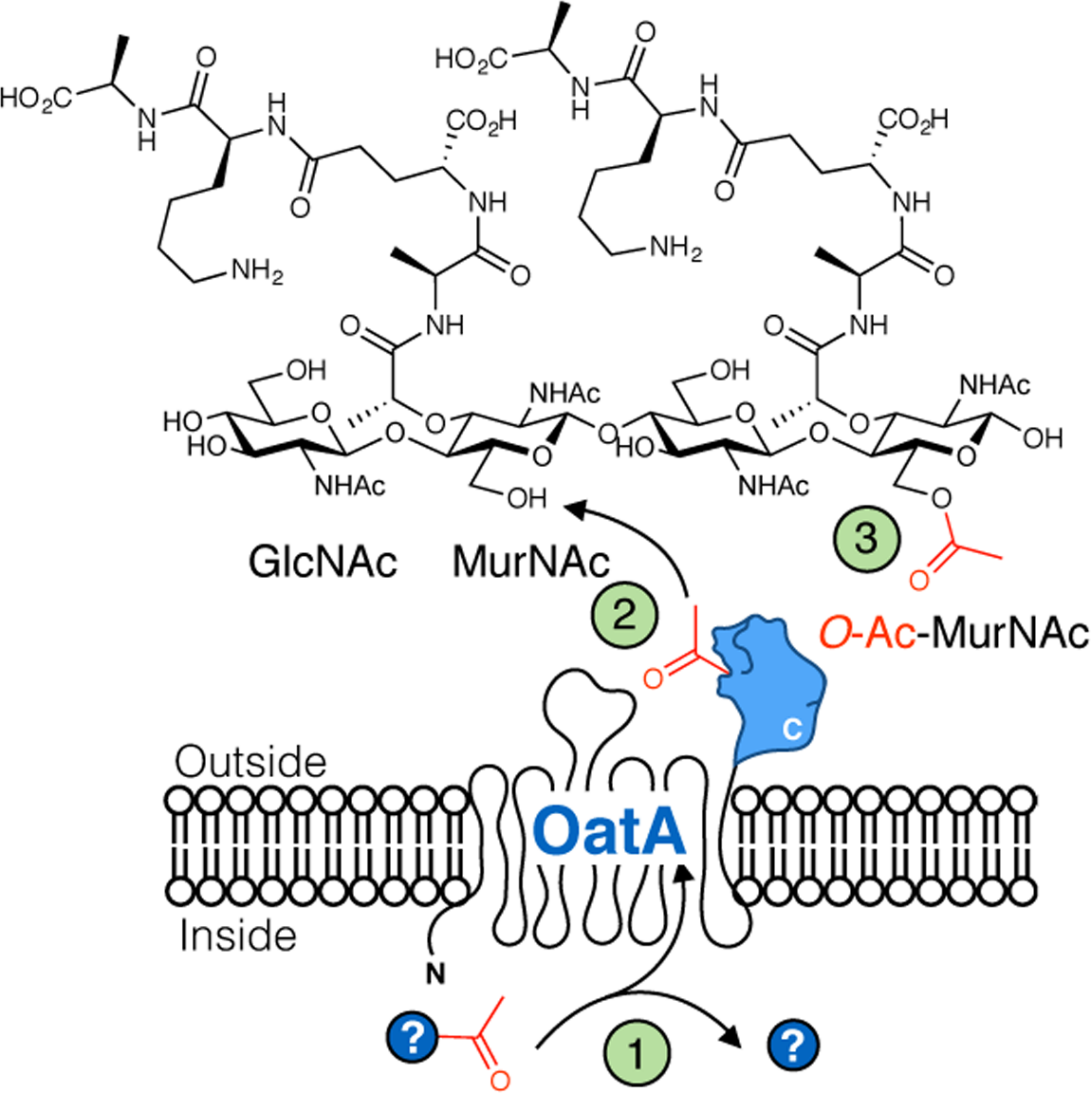
Proposed pathway for PG O-acetylation by OatA. (1) An acetyl group from an unidentified donor is obtained from the cytoplasm by the N-terminal domain of OatA and then it is translocated across the inner membrane. (2) The extracytoplasmic C-terminal domain of OatA accepts the acetyl group and catalyzes the acetyltransfer to modify the C6-OH of MurNAc residues within PG. (3) The final product: 6-*O*-acetyl-MurNAc.

The likely natural source of the acetyl groups for PG O-acetylation, acetyl-CoA, does not serve as a donor substrate for OatA_C_. Given this, we postulate that the putative membrane-spanning, N-terminal OatA domain functions like PatA of Gram-negative bacteria [6,8,29] to translocate acetyl groups from a cytoplasmic source, presumably acetyl-CoA, across the membrane for their transfer to PG by OatA_C_ (**Fig 8**). Whether the acetyl group is transferred directly from the N-terminal membrane domain to OatA_C_ or *via* an exogenous carrier has yet to be determined. It is also not clear whether or not the two domains remain attached as a single bimodular protein following translation and insertion into the cytoplasmic membrane. Indeed, *S*. *aureus* OatA possesses a non-canonical type I signal peptidase cleavage site between the two domains, and the C-terminal domain alone has been detected in spent culture media [45].

**Fig 9:**
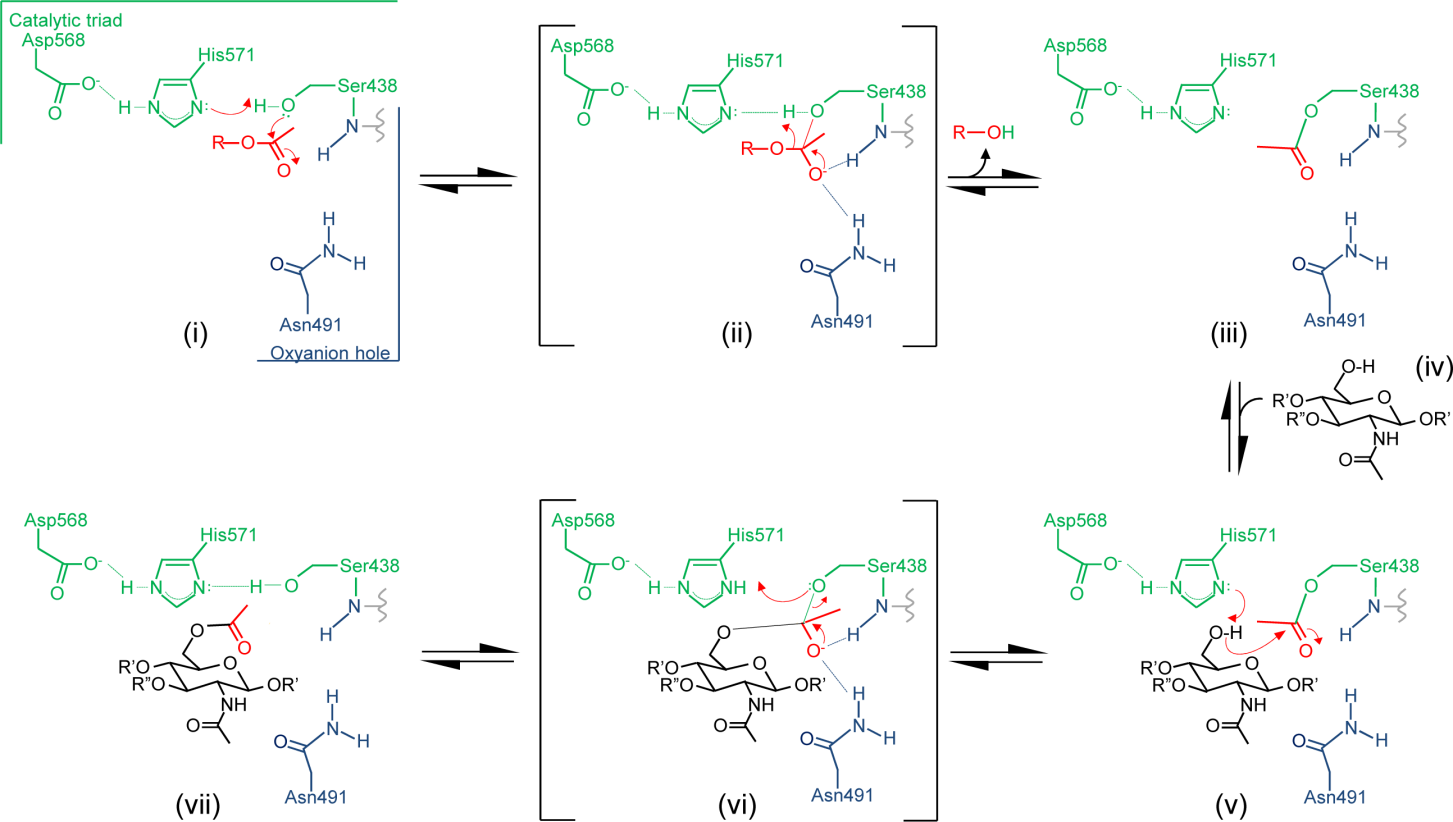
Proposed mechanism of action of OatA. (i) An acetyl donor binds into the active site and causes Asn491 to align appropriately within the oxyanion hole. The H-bonding network between the catalytic triad residues increases the nucleophilicity of the Ser438 hydroxyl which attacks the carbonyl center of the bound acetyl group. (ii) The putative transient tetrahedral oxyanion intermediate is stabilized by the backbone amide of Ser438 and side-chain amide of Asn491. (iii) An acetyl-enzyme intermediate forms with the departure of the donor group. (iv) A MurNAc residue on PG binds, possibly involving Asn491, and (v) His571 serves as a base to abstract the proton from the C6 hydroxyl group to permit its nucleophilic attack on the carbonyl center of the acetyl-enzyme. (vi) The second transient oxyanion intermediate formed is stabilized by the oxyanion hole residues prior to its (vii) collapse releasing the 6-*O*-acetylPG product. Not depicted are the transition states that lead to and from each of the oxyanion intermediates. R, the acetyl donor molecule; R’, GlcNAc residues of a PG glycan chain; R”, lactyl group and associated stem peptide.

Considering the kinetic and structural data presented above, we propose that OatA_C_ employs a double-displacement mechanism of action involving a covalent acetyl-enzyme intermediate (**Fig 9)**. As we have shown that the enzyme exists in both a resting and catalytically-active state, we suggest that binding of an acetyl donor molecule induces a conformational change involving the side chain of Asn491 to form the oxyanion hole which, together with the backbone amide of Ser438, would serve to: (i) increase the electrophilicity of the carbonyl C to facilitate nucleophilic attack by the Ser438 hydroxyl group, and (ii) stabilize the negatively charged oxyanion of the tetrahedral transition state [46]. The nucleophilic attack on the carbonyl carbon of the acetyl donor by the Oδ of Ser438 is aided by abstraction of its proton by His571 and leads to a tetrahedral oxyanion, which is stabilized by the oxyanion hole residues Ser438 and Asn491 (Figs 6 and 7). The oxyanion collapses to the covalent acetyl-enzyme intermediate concomitant with the release of the donor product. A MurNAc residue of a PG glycan strand would then bind into the active site cleft and His571 again functions as a base to abstract the proton from the C6 hydroxyl group of the acceptor and render it nucleophilic. Attack by this C6 alkoxide on the carbonyl center of the acetyl-Ser438 leads to the formation of a second tetrahedral oxyanion, which then collapses to generate the *O*-acetyl MurNAc.

Previous phylogenetic analysis of OatA had shown that homologs are distributed between three distinct clades [28], where each cluster includes primarily proteins from a single bacterial order (Lactobacillales and Bacillales). The exception is the genus *Streptococcus* (belonging to the Lactobacillales) for which the homologs of this group have branched into their own clade. Until now, only the two consensus motifs GDSV and Dx(I/V)H harboring the predicated catalytic triad residues had been identified in the C-terminal domain of OatA [28,47]. Our structural and biochemical characterization of *Sp*OatA_C_ demonstrated experimentally the functional significance of these conserved residues. More importantly, it enabled the identification of two additional motifs, the **G**(T/V)**N** motif containing Asn491 as an H-bond donor to the oxyanion hole, and the (**V**/I)(**G**/S)(**R**/V) motif as part of the type-I β-turn in the Block II-loop (**Fig 6B**). While lacking the signature Gly of the oxyanion holes in SGNH/GDSL hydrolases, the retention of Asn in OatA signifies its closer evolutionary relationship to these enzymes compared to the PC-Esterases, a GDSL family of bimodular enzymes in eukaryotes that modify extracellular matrices; the PC-esterases lack both the Gly and Asn residues [48].

Replacement of Asn491 with Ala abolished transferase activity while reducing the specific activity of *Sp*OatA_C_ as an esterase toward *p*NP-Ac by only 58% (**Table 1**). Whereas this finding is consistent with our assignment of Asn491 as comprising the oxyanion hole for the stabilization of the transition state leading to formation of O-acetylated product, the level of residual esterase activity would suggest that Asn491 does not contribute significantly to the stabilization of the transition state for the first half of the reaction pathway involving the generation of the acetyl-enzyme. However, it should be recognized that enzyme-catalyzed hydrolysis of *p*NP-Ac proceeds through a transition state that has some tetrahedral character while maintaining partial carbonyl π bonding [49], thus reducing the need for oxyanion hole stabilization. Also, it is possible that in addition to comprising the oxyanion hole, Asn491 contributes to the productive binding of acetyl-acceptor glycans. In this regard, examination of the surface topology of *Sp*OatA_C_ (**Fig 5B**) does not reveal a deep active site pocket/cleft. Moreover, unlike most carbohydrate-active enzymes, the shallow putative substrate-binding pocket is devoid of any aromatic residues. Despite lacking these common features, OatA_C_ is active as a transferase on only glycans with a DP ≥4 (**Figs 2 and 3**) suggesting that the enzyme possesses at least four carbohydrate-binding subsites. Presumably, these are arranged on its surface and serve to position specifically the C6 hydroxyl group of MurNAc residues for O-acetylation.

Whereas OatA_C_ has the capacity to function as an esterase *in vitro*, this hydrolytic activity of the extracytoplasmic domain would need to be minimized, if not precluded, *in vivo* to prevent the wasteful loss of acetyl groups (as acetate) to the external milieu. It is likely that a water-limiting environment is created by the juxtaposition of the domain with both the cytoplasmic membrane and the insoluble PG sacculus though this alone would not be sufficient to preclude water access. Jiang *et al*. [50] have observed that the type of β turn of the loop harboring an oxyanion residue distinguishes between hydrolytic and acyltransferase activities in some classical α/β hydrolases. Their observations invoke the participation of a bridging water that is H-bonded to the main-chain of a residue in the β-turn which either activates (type-II) or deactivates (type-I) the attacking water to promote hydrolase or transferase activity, respectively. However, our analysis of the SGNH/GDSL hydrolases and *Sp*OatA_C_ did not identify a bridging water molecule. Therefore, the consequences of the β-turn differences observed with a subset of the classical serine esterases/acyltransferases does not appear to apply to the SGNH/GDSL enzymes.

More recently, Light *et al*. [51] have proposed that substrate binding to non-catalytic domains combined with a conformationally-stable active site promote transfer reactions, whereas conformational change at the active site is associated with hydrolysis. Their observations were made with glycosyl transferases, but it is possible that the same principles apply to esterases/transferases with homologous structures. With *Sp*OatA_C_, we are unable to assess such binding contributions to its reaction pathway because the structure of the binding sub-sites and their associated interactions PG remains unknown. However, we did find through bioinformatic and protein engineering studies the importance of an invariant Val or Ile residue at the active site of the enzyme for transferase activity (Val490 in *Sp*OatA_C_). Presumably, either of these residues serve to stabilize acceptor substrates through hydrophobic interactions between their alkyl side-chains and the hydrophobic patches associated with carbohydrates. Also, examination of the MeS adduct (a mimic of the carbonyl O of a bound acetyl group) depicted in **Fig 7** suggests the approach of an acceptor ligand (*e*.*g*., water or a carbohydrate) to the carbonyl C of the bound acetyl group would have to be from a hydrophobic pocket formed by Val490 and Val570. Thus, it is possible that the relative hydrophobicity of the reaction centre helps to restrict hydrolysis (esterase activity) and/or promote efficient transferase activity.

As a maturation event, the O-acetylation of PG occurs extracytoplasmically on the existing PG sacculus [6,7,43]. Earlier biochemical studies involving pulse-chase experiments suggested the timing of the modification varies with species. Our kinetic characterization of OatA_C_ from *S*. *aureus* and *S*. *pneumoniae* now provides a plausible explanation for this temporal difference at the molecular level. The final stages of PG biosynthesis involve the transglycosylation of Lipid II precursors into the growing glycan strand which is followed by the crosslinking of neighboring stem peptides. The latter occurs through a transpeptidation reaction whereby the crosslink is made with the concomitant loss of the terminal d-Ala from the donating stem pentapeptide, resulting in the formation of tetrapeptide stems. As *Sa*OatA_C_ has a high specificity for PG glycan chains possessing pentapeptide stems (muroglycan-5P; Fig 3), O-acetylation in this bacterium would have to immediately follow the transglycosylation reaction and precede transpeptidation. This is indeed consistent with the early observations of Snowden *et al*. [52] who suggested that O-acetylation must be very closely linked with the addition of PG units to the growing polymer. An analogous specificity would appear to exist in vancomycin-resistant *E*. *faecailis* where muropentapeptides terminating with d-Ala-d-Lac residues were found recently to be preferentially O-acetylated [53]. *Sp*OatA_C_, on the other hand, has specificity for muroglycans with tetrapeptide stems (**Fig 3**) and hence it would require the prior crosslinking and/or processing of nascent PG by a d,d-carboxypeptidase such as DacA before it could act. Further processing of stem peptides by a d,l-carboxpeptidase such as DacB to generate GM-tripeptide repeats would preclude continued O-acetylation. These unique specificities explain why, unlike PatB of Gram-negative bacteria [29,30], OatA_C_ was able to O-acetylate muroglycans derived from natural sacculi where the PG has undergone maturation. Thus, in addition to controlling the degree of crosslinking [54], it would appear that the activity of carboxypeptidases such as DacA and DacB provides a means of control of PG O-acetylation at the substrate level.

Another level of control of OatA activity imposed at the substrate level may concern its localization within a given species. The transglycosylation reactions for PG biosynthesis are catalyzed by the Class A PBPs [55], and mono-functional transglycosylases [56–58]. The Class A PBPs are bifunctional possessing both transglycosylase and transpeptidase activities [55]. Consequently, OatA in *S*. *aureus* would need to be positioned in close proximity to, if not complexed with, one or both of its mono-functional transglycosylases so that it may act on the newly incorporated Lipid II precursors while they still possess their stem pentapeptides. Nothing is known about the organization of OatA and the monofunctional transglyosylases in *S*. *aureus*, but *L*. *plantarum* OatA was found to play a key role in the spatio-temporal control of cell elongation and septation. This function of OatA does not require its catalytic activity as an *O*-acetyltransferase [27], suggesting that the protein helps to coordinate the PG biosynthetic complex of enzymes in PG metabolism. Hence, it is conceivable that *S*. *aureus* OatA may indeed complex with one or both of the monofunctional transglycosylases to permit PG O-acetylation. With *S*. *pneumoniae*, on the other hand, OatA would need to remain free and/or associated with its bi-functional PBPs for it to act on PG subunits with tetrapeptide stems following crosslinking reactions.

This report has addressed several important aspects of PG O-acetylation in two human pathogens that have already overburdened healthcare systems worldwide. OatA is a key enzyme involved in bacterial resistance to the human innate immune response and it has been suggested to represent a useful target for pharmacological intervention [59], which may apply especially to the treatment of MRSA and VRE. Our discovery that *Sa*OatA_C_ and *Sp*OatA_C_ have different substrate specificities will be an important consideration in the development of novel inhibitors that may serve as antivirulence agents for the sensitization of Gram-positive pathogens containing *O*-acetyl-PG to the lysozymes of innate immunity systems. In addition, this work will aid in the characterization of other carbohydrate *O*-acetyltransferases predicted to contain an SGNH hydrolase fold that perform important physiological and pathological roles in organisms from other kingdoms of life [60,61].

## Materials and methods

### Cloning, engineering, and production of *Sp*OatA_C_ and *Sa*OatA_C_

The gene sequences encoding the extracytoplasmic domains of OatA from *S*. *aureus* and *S*. *pneumoniae* were identified based on topology and fold predictions using HHMTOP [62] and Phyre2 [63], respectively. The gene encoding *Sa*OatA_C_ (residues 435–603) was amplified by PCR using genomic DNA from *S*. *aureus* SA113 and the codon-optimized gene encoding *Sp*OatA_C_ (residues 423–605; originally from *S*. *pneumoniae* R6) was synthesized and provided in a pUC57 (pUC57-*Sp*OatA_C_) vector from Genscript (Piscataway, NJ). For cloning, the PCR product encoding *Sa*OatA_C_, pUC57-*Sp*OatA_C_, and the expression vector pBAD-His A (Invitrogen, Burlington, ON) were digested with *Xho*I and *Eco*RI. Each gene was then ligated to pBAD-His A to produce pACPM31 (*Sa*OatA_C_) and pDSAC81 (*Sp*OatA_C_). Both constructs contain an enterokinase-cleavable N-terminal His_6_ tag and are under the control of an arabinose inducible promoter. Site-specific replacements of amino acid residues for both constructs were performed by site-directed mutagenesis using the QuickChange Site-Directed Mutagenesis Kit (Agilent Technologies Canada Inc., Mississauga, ON) with the appropriate primers listed in **S2 Table**.

For the production of *Sa*OatA_C_ or *Sp*OatA_C_, *Escherichia coli* BL21 (DE23) was transformed with pACPM31 or pDSAC81, respectively. Cells were grown in LB broth containing 100 μg·mL^-1^ ampicillin at 37 ^o^C until an OD_600_ of 0.6 was reached, at which point arabinose was added to a final concentration of 0.2% (*w/v*). The cultures continued to grow at 37 ^o^C for an additional 4 h, after which the cells were harvested by centrifugation (5, 000 × *g*, 4 ^o^C, 15 min) and frozen at—20 ^o^C until needed. For the production of selenomethionine-labelled (SeMet) *Sp*OatA_C_, pDSAC81 was transformed into *E*. *coli* B834 and grown in M9 minimal media supplemented with 40 mg selenomethionine as previously described [64]. The expression of SeMet-*Sp*OatA_C_ was carried out as described above for native protein.

To purify *Sa*OatA_C_, the cell pellets were resuspended in lysis buffer (50 mM sodium phosophate buffer, pH 7.8, 500 mM NaCl, 20 mM imidazole, 20 μg·mL^-1^ DNase, 20 μg·mL^-1^ RNase, and 50 μg·mL^-1^ hen egg-white lysozyme) and disrupted by sonication on ice. Unbroken cells were cleared from the lysate by centrifugation (15,000 *x g*, 4 ^o^C, 15 min) and the supernatant was incubated with cOmplete His-Tag purification resin (Roche Diagnostics, Laval, QC) pre-equilibrated with wash buffer (50 mM sodium phosphate pH 8.0, 500 mM NaCl). After 1 h at 4 ^o^C with nutation, the cell lysate containing *Sa*OatA_C_-bound resin was loaded onto a gravity-flow column. The resin was washed with 100 mL wash buffer and then *Sa*OatA_C_ was eluted using wash buffer containing 250 mM imidazole. Following elution, *Sa*OatA_C_ was dialyzed against 25 mM sodium phosphate buffer 6.5 at ambient temperature for 1 h (with one buffer change). The dialyzed protein was filtered using a syringe driven filter (0.22 μm; Milipore) and loaded onto a Source 15S cation-exchange column (GE Health Care Canada Inc., Mississauga, ON) pre-equilibrated with dialysis buffer using an NGC protein purification system (Bio-Rad Laboratories (Canada) Ltd, Mississauga, ON). Protein elution was achieved with a linear gradient of 0−1 M NaCl at a flow-rate of 1 mL·min^-1^.

*Sp*OatA_C_ was purified similarly, however, in all cases the phosphate buffer was substituted for Tris-HCl buffer, dialysis was performed at pH 8.0, and anion-exchange chromatography was conducted using a Source 15Q column (GE Health Care) at pH 8.0.

The production and purification of *Sa*OatA_C_ and *Sp*OatA_C_ possessing site-specific amino acid replacements were performed as described above, respectively, with the precaution of using fresh chromatography media to preclude the possibility of contamination with wild-type enzymes. The secondary structure of each purified protein was assessed by circular dichroism (CD) spectroscopy to ensure their correct folding.

### Cloning, production, and purification of DacA and DacB

The genes encoding DacA (covering residues 23–394) and DacB (covering residues 56–238) lacking both their N-terminal trans-membrane and C-terminal membrane interaction helices were PCR amplified from *S*. *pneumoniae* R6 genomic DNA with the primers listed in **S2 Table.** Both PCR products were digested with *Nde*I and *Xho*I and ligated into pET-28a. The resulting constructs, pDSAC01 and pDSAC02 harboring *dacA* and *dacB* respectively, contained each gene in frame with an N-terminal His_6_ tag under the control of an IPTG inducible promoter.

For the overproduction of DacA or DacB, the respective plasmids were transformed into *E*. *coli* BL21 pLysS and *E*. *coli* T7 Shuffle, respectively. The cells were grown in LB broth supplemented with 50 μg·mL^-1^ kanamycin until an OD_600_ of 0.6 was reached, at which point expression was induced with IPTG at a final concentration of 1 mM. After 4 h of additional growth, the cells were harvested by centrifugation (5000 × *g*, 15 min, 4 ^o^C) and the cell pellets were frozen at -20 ^o^C until needed. For purification, the cells were lysed by sonication in lysis buffer (50 mM Tris-HCl pH 8.0, 500 mM NaCl), the His_6_-tagged proteins were bound to cOmplete His-Tag purification resin, and eluted with lysis buffer containing 300 mM imidazole as described previously [31]. The purified proteins were dialyzed into 50 mM Tris-HCl pH 8.0 and kept at -20 ^o^C until required.

### CD spectroscopy

*Sp*OatA_C_, *Sa*OatA_C_, and their variants were diluted to 0.15 mg·mL^-1^ in 10 mM sodium phosphate buffer pH 7.0. CD spectra (190 nm—260 nm, 1 nm increments) of samples in a 0.1 cm quartz cuvette were measured in triplicate at 25 ^o^C using a Jasco Model J-815 CD spectrometer (Jasco Inc., Easton, MD).

### Enzyme assay and reaction product analysis

To identify a suitable acetyl-donor for the OatA_C_ homologs, 100 μL reaction mixtures containing enzyme (5 μM), 1 mM donor (acetyl-CoA, *p*NP-Ac, or 4MU-Ac) and 2 mM chitotetraose in 25 mM sodium phosphate buffer pH 6.5 were incubated at 37 ^o^C for 1 h. Reactions were terminated by separating the substrates from the enzyme using porous graphitized carbon (PGC) solid-phase extraction (SPE) cartridges, previously charged with acetonitrile (ACN) and equilibrated with water. The PGC-SPE cartridges were washed with three volumes of water and both chitotetraose and *O*-acetylated products were eluted with 0.5 mL ACN/water (1:1). ESI-MS analysis was performed by direct infusion using an Amazon SL ion-trap mass spectrometer (Bruker Daltonics Ltd., Milton, ON) at a flow rate of 5 μL·min^-1^ with a spray voltage of 4.5 kV. The ion-trap was operated in positive ion mode and MS scans ranging from 200–2200 *m/z*. MS/MS scans were made on the major ions with a fragmentation amplitude of 1.0. Mass spectra were analyzed using Bruker Compass tool (Bruker).

### pH dependence of *p*NP-Ac hydrolysis

The pH optima for the *Sp*OatA_C_- and *Sa*OatA_C_-catalyzed hydrolysis of *p*NP-Ac were determined using the spectrophotometric assay for *p*NP release as described by Moynihan and Clarke [31]. Triplicate reaction mixtures (200 μL) contained 5 μM enzyme and 1 mM *p*NP-Ac in 25 mM sodium borate-phosphate-citrate buffer with pH values ranging from 5 to 7.5 with 0.5 unit intervals. Hydrolysis was monitored at 410 nm over 15 min at 25 ^o^C and enzymatic rates were determined by subtracting the rates of spontaneous *p*NP-Ac hydrolysis of control reactions lacking enzyme.

### Steady-state reaction kinetics of hydrolysis and acetyltransfer

The determination of steady-state Michaelis-Menten parameters for enzyme-catalyzed hydrolysis of *p*NP-Ac (esterase activity) were made using the spectrophotometric assay described above. Initial rates of 0.005–5 mM *p*NP-Ac hydrolysis in 50 mM sodium phosphate buffer pH 6.5 containing 5% (*v/v*) ethanol (to maintain solubility of substrate) were determined following the addition of 5 μM *Sp*OatA_C_ or 3 μM *Sa*OatA_C_ (final concentration). The steady-state kinetics of acetyltransfer catalyzed by *Sp*OatA_C_ were determined also using the spectrophotometric assay for *p*NP release [38]. Enzyme (5 μM) in 50 mM sodium phosphate buffer pH 6.5 was incubated at 25 ^o^C with 2 mM *p*NP-Ac and varying concentrations of chito-oligosaccharides (DP 3–6) in a total volume of 150 μL. Control reactions lacked the chito-oligosaccharide acceptors. The net rate of acetyltransfer was determined by subtracting the initial rates of *p*NP release in control reactions from reactions containing chitooligosaccharides. The Michaelis-Menten kinetic parameters were determined by non-linear regression using GraphPad Prism 4 (GraphPad Software, Inc., La Jolla, CA). Each of these experiments was performed with three different preparations of the enzymes.

### Inactivation of *Sp*OatA_C_ by MSF

Enzyme (25 μM) in 25 mM sodium phosphate buffer pH 6.5 was incubated with 5 mM MSF at 25 ^o^C for 20 min. Samples were withdrawn and assayed for *p*NP-Ac hydrolytic activity as described above.

### Preparation of linear muroglycans

Lys-containing Lipid II (partially labeled with Dansyl chloride for facile detection) was prepared enzymatically as previously described [33]. Linear muroglycans were generated by polymerizing Lipid II in 50 mM HEPES buffer pH 7.5, 200 mM NaCl, 25 mM MgCl, 25% (*v/v*) DMSO, and varying concentrations of Triton X-100 using soluble *S*. *pneumoniae* PBP2a (covering residues 78–731) lacking its N-terminal transmembrane helix. Following incubation at 30 ºC overnight, the reaction mixture was heat inactivated at 90 ^o^C for 10 min and precipitated PBP2a was removed by centrifugation (10,000 × *g*, 5 min). The DP was determined by SDS PAGE analysis with fluorescence detection of the Dansyl label using 8.5% acrylamide gels [65].

Muroglycans with tetra- and tri-peptides were prepared by incubation of the original PBP2a product with recombinant d,d-carboxypepdiase DacA and l,d-carboxypeptidase from *S*. *pnuemonae* R6. Samples (100 μL) of the muroglycans (15 μg·mL^-1^) in 50 mM sodium phosphate buffer pH 6.5 were incubated at 37 ^o^C overnight with DacA alone or with both DacA and DacB (final concentration of each enzyme, 5 μM). Heat inactivation at 95 ^o^C for 10 min was used again to quench further reaction and precipitated protein(s) was removed by centrifugation (10,000 × *g*, 5 min).

### Specificity of *Sa*OatA_C_ and *Sp*OatA_C_ for muroglycans

Enzyme (10 μM) in 50 mM sodium phosphate buffer pH 6.5 was incubated at 37 ^o^C for 1 h with 0.5 mM *p*NP-Ac, and 10 μg·mL^-1^ muroglycans possessing either penta, tetra, or tripeptide stems. The enzymes were heat inactivated at 95 ^o^C for 30 min and then removed by centrifugation (10,000 × *g*, 5 min). The PG oligomers were digested overnight at 37°C with 100 μg·mL^-1^ mutanolysin which was added directly to the reaction product pool. Digestion of the muroglycans to monomers (GM-peptide) was necessary for detection of the reaction products by ESI-MS. Following digestion, the reaction products were subjected to adsorption chromatography on PGC-SPE as described above, except the elution solvent contained 0.1% formic acid to facilitate the desorption of the charged muropeptides. Reaction products were then analyzed by ESI-MS. These experiments were repeated once with identical results.

### Crystallization of *Sp*OatA_C_

*Sp*OatA_C_ was concentrated to 42 mg·mL^-1^ using an Amicon Ultra-15 centrifugal filter (30 kDa MWCO; Millipore (Canada) Ltd., Etobicoke, ON) at 4,000 × *g* and 4 ^o^C, followed by centrifugation (15,000 × *g*, 10 min, 4 ^o^C) to remove any insoluble material. The concentrated protein sample was used in the MCSG Crystallization Suite sparse matrix crystallization screens 1 to 4 (Microlytic North America Inc., Burlington, MA). Crystallization screening using the sitting drop vapor diffusion method was setup using a Gryphon robot (Art Robbins Instruments, Sunnyvale, CA) with 1 µL drops of protein and a protein to reservoir ratio of 1:1. Large single diffraction quality crystals appeared after one week of incubation at 21 ^o^C in 0.1 M HEPES:NaOH pH 7.5, 1.2 M sodium citrate tribasic; and 2.4 M sodium malonate pH 7. Crystal screening of selenomethionine (SeMet) labeled OatA was carried out as described above and large single crystals were grown in 2.4 M sodium malonate pH 7. To produce *Sp*OatA_C_ in complex with MeS, crystals grown in 1.8 M NaH_2_PO_4_/K_2_HPO_4_, pH 8; 0.1 M HEPES:NaOH pH 7.5, 1.4 M sodium citrate tribasic were soaked in mother liquor containing 1.2 M sodium citrate tribasic and 250 mM MSF for 24 hours.

### X-ray diffraction data collection and structure determination

Crystals were cryoprotected for 5–10 s in reservoir solution supplemented with 25% (*v/v*) ethylene glycol prior to vitrification in liquid nitrogen. Native and selenium single-wavelength anomalous diffraction (Se-SAD) data were collected on beam line X29 at the National Synchrotron Light Source (Upton, NY) (S1 Table). The data were indexed and scaled using HKL2000 [66]. The Se-SAD data were used in conjunction with HKL2MAP [67] to locate four selenium sites, and density modified phases were calculated using SOLVE/RESOLVE [68]. The resulting electron density map was of good quality and enabled PHENIX AutoBuild [69] to build 100% of the protein. Manual model building of the remaining residues was completed in COOT [70] and alternated with refinement using PHENIX.REFINE [71]. The structures of the native and MeS proteins were determined by molecular replacement using the SeMet incorporated derivative and the native structure as the search model. The PHENIX AutoMR algorithm [71] was used with manual model building and refinement carried out as described previously. Translation/ Libration/Screw groups were used during refinement and determined automatically using the TLSMD web server [72,73]. All molecular models were generated using Pymol and structural superpositions were made using the cealign plug-in.

### Other analytical procedures

Nucleotide sequencing of PCR products, as well as plasmids, was performed by the Genomics Facility of the Advanced Analysis Center (University of Guelph). Protein concentrations were determined using the Pierce BCA protein assay kit (Pierce Biotechnology, Rockford, IL) with BSA serving as the standard. SDS-PAGE on 15% acrylamide gels was conducted by the method of Laemmli [74] with Coommassie Brilliant Blue staining and Western immunoblot analysis as previously described [29].

Data deposition: The crystallography, atomic coordinates, and structure factors have been deposited in the Protein Data Bank, www.pdb.org (PDB ID code 5UFY (*Sp*OatA_C_); 5UG1 (*Sp*OatA_C_MeS).

## Supporting information

S1 Table Crystal structure data collection and refinement statistics. (PDF)

S2 Table List of oligonucleotide primers used in this study. (PDF)

S1 Fig Purification of *Sp*OatA_C_ and *Sa*OatA_C_. **A**. SDS-PAGE analysis of the purification of *sp*OatA_C_. Lanes: 1, Elution fraction from His-Tag purification resin; 2 and 3, Fractions 1 and 3 of SourceQ elution, respectively. **B**. SDS-PAGE analysis of the purification of *Sa*OatA_C_. Lanes 1, 2, and 3, correspond to the same purification steps as described in panel **A**.
(TIF)

S2 Fig MS/MS fragmentation spectra of *O*-acetyltransferase reaction products. The MS/MS fragmentation spectra of **A**, the sodiated di-*O*-acetyl-chitotetraose parent ion (937.33 [M+Na]^+^) produced by *Sp*OatA_C_ and **B**, the sodiated *O*-acetyl-chitotetraose parent ion (895.35 [M+Na]^+^) produced by *Sa*OatA_C_. Both sodium adducts were generated from the respective protonated species with 0.1 mM NaCl to facilitate the cross-ring cleavages. **C** and **D**, Interpretation of the fragment ions presented in panels **A** and **B**, respectively.
(TIF)

S3 Fig Continuous assay for *O*-acetyltransferase activity of *Sp*OatA_C_ and *Sa*OatA_C_. Progress curves of *p*NP release from 1 mM *p*NP-Ac in 50 mM sodium phosphate buffer pH 6.5 incubated at 25 ºC with **A**, *Sp*OatA_C_ and **B**, *Sa*OatA_C_ in the absence (red) and presence (blue) of 2 mM chitopentaose. The spontaneous release of *p*NP from *p*NP-Ac incubated under the same conditions but without added enzyme is represented by the black symbols. Each assay was performed in triplicate, with the s.e. noted.
(TIF)

S4 Fig ESI-MS analysis of *Sp*OatAC-catalyzed *O*-acetyltransferase reaction products. Enzyme (5 μM) in 50 mM sodium phosphate buffer pH 6.5 was incubated for 1 h at 37 ºC with 1 mM *p*NP-Ac and 2 mM **A**, chitopentaose (G5; [M+H/Na]^+^) and **B**, chitohexaose (G6; [M+H/Na/K]^2+^). Reaction products were isolated by adsorption to PGC solid-phase extraction cartridges prior to ESI-MS analysis 7by direct infusion using an Amazon SL ion-trap mass spectrometer.
(TIF)

S5 Fig Structure of muroglycan-5P (DP 2–8). (TIF)

S6 Fig Polymerization lipid II by *S*. *pneumoniae* PBP2a. SDS PAGE analysis with fluorescence detection of reaction products of partially Dansylated lipid II (10 μM) in 50 mM HEPES buffer pH 7.5 containing 200 mM NaCl, 25 mM MgCl, 25% (v/v) DMSO, and varying concentrations of Triton X-100 as indicated in the absence and presence of incubated overnight at 30 ºC with *S*. *pneumoniae* PBP2a.
(TIF)

S7 Fig Electron density maps of the *Sp*OatA_C_ active site. The 2m*F*_*o*_-D *F*_*c*_ maps (gray) of the active sites of **A**, native *Sp*OatA_C_ and **B**, *Sp*OatA_C_-MeS are contoured at 1.0 σ. The m*F*_*o*_-D *F*_*c*_ omit map of the MeS-Ser438 adduct (green) is contoured at 3.0 σ.
(TIF)

S8 Fig Amino acid alignment of the Block II sequences in known and hypothetical OatA homologs. The sequence of *S*. *aureus* OatA was used as the query in a TBLASTN search of the completed bacterial genomes. The residues in bold face and highlighted in yellow denote greater than 50% and 80% identity, respectively, while invariant the Val/Ile at position 5 of the Block are in red. Only the sequence of a representative strain of the species listed is presented.
(TIF)
