# Supplementary material for: In vitro characterization of the antivirulence target of Gram-positive pathogens, peptidoglycan O-acetyltransferase A (OatA)
Source: PLoS Pathog. 2017 Oct 27;13(10):e1006667. doi: 10.1371/journal.ppat.1006667 (PMC5697884; doi:10.1371/journal.ppat.1006667)
Supplement: S1 Table — (PDF) [file ppat.1006667.s001.pdf]

Table S1. Crystal structure data collection and refinement statistics

	Native $SpOatA_C$	$SpOatA_C$MeS	$SpOatA_C$ SeMet
Data collection			
Beamline	NSLS X29	Home source	NSLS X29
Wavelength (Å)	1.08	1.54	0.979
Space group	*I*422	*I*422	*I*422
Unit cell parameters (Å)	*a = 70.2*	*a = 70.5*	*a = 68.6*
	b = 70.2	*b = 70.5*	*b = 68.6*
	c = 135.6	*c = 136.2*	*c = 136.7*
	α = β = γ = 90°	α = β=γ = 90°	α = β=γ = 90°
Resolution (Å) range	50.00 - 1.12	25.9 - 2.10	50.00 - 1.80
max.	1.16 - 1.12	2.17 -2.10	1.86 - 1.80
Total no. reflections	1885423	136218	425144
No. unique reflections	65286	101091	15505
Redundancy	29.1 (26.2)	13.4 (11.4)	27.5 (28.4)
Completeness (%)	99.1 (97.6)	98.0 (94.6)	99.8 (99.9)
Average *I*/σ*I*	64.2 (13.2)	11.8 (4.0)	76.5 (6.0)
R_{merge} (%)[1]	5.3 (34.4)	14.4 (50.2)	8.7 (68.9)
Refinement			
R_{work}/R_{free}[2]	15.0 / 16.8	17.2 / 23.5	
No. of atoms	1622	1523	
Protein	1622	1362	
Water	243	156	
Ligand	1	5	
Average *B*-factor ($Å^2$)[3]	17.6	29.5	
Protein	15.30	28.8	
Water	30.8	35.0	
RMS deviations			
Bond lengths (Å)	0.006	0.007	
Bond angles (◦)	0.94	0.75	
Ramachandran plot[3]			
Total favored (%)	97	96	
Total allowed (%)	100	100	
Coordinate error (Å)[4]	0.09	0.26	
PDB entry	5UFY	5UG1	

[1]R_{merge}= ∑∑ |(k0 - <*I* > |/∑*I*(k) where *I* (k) and <*I*> represent the diffraction intensity values of the individual measurements and the corresponding mean values. The summation is over all unique measurements.

[2]R_{work}= ∑||F_{obs}| - k|F_{calc}|| / |F_{obs}| where F_{obs} and F_{calc} are the observed and calculated structure factors, respectively. R_{free} is the sum extended over a subset (5%) of reflections excluded from all stages of the refinement.

[3]As calculated using MolProbity [75].

[4]As calculated by PHENIX [69].
