# Supplementary material for: In vitro characterization of the antivirulence target of Gram-positive pathogens, peptidoglycan O-acetyltransferase A (OatA)
Source: PLoS Pathog. 2017 Oct 27;13(10):e1006667. doi: 10.1371/journal.ppat.1006667 (PMC5697884; doi:10.1371/journal.ppat.1006667)
Supplement: S2 Table — (PDF) [file ppat.1006667.s002.pdf]

Name	Sequence (5' →3')	Description	Plasmid
OatAC3$_{Fwd}$	CGAGCTCGAGGATAAGCAAGAGGATAAG	Forward primer containing a *Xho*I site for cloning *oatA$_C$* from *S. aureus* (residues 435 - 603) into pBAD-HisA	pACPM31
OatAC$_{Rev}$	CAGCCGAATTCTTATTTCTTATTTGTAGCATGTG	Reverse primer containing an *Eco*RI site for cloning *oatA$_C$* from *S. aureus* (residues 435 - 603) into pBAD-HisA	pACPM31
OatA-S453A$_{Fwd}$	GGTGACGCGGTCATGGTGGATATTG	Forward primer for site-directed mutagenesis of pACPM31 to produce a S453A replacement.	pACLK31-S
OatA-S453A$_{Rev}$	CCACCATGAACCGCGGCTAA	Reverse primer for site-directed mutagenesis of pACPM31 to produce a S453A replacement.	pACLK31-S
OatA-H579A$_{Fwd}$	GACGGTATTGCGTTAGAATATGCAG	Forward primer for site-directed mutagenesis of pACPM31 to produce a H579A replacement.	pACLK31-H
OatA-H579A$_{Rev}$	TTCTAACGCAATACCGTCATATGCAA	Reverse primer for site-directed mutagenesis of pACPM31 to produce a H579A replacement.	pACLK31-H
OatA-D575A$_{Fwd}$	GCATATGCGGGTATTCACTTAG	Forward primer for site-directed mutagenesis of pACPM31 to produce a D575A replacement.	pACLK31-D
OatA-D575A$_{Rev}$	GAATACCCGCATATGCAAAGTAT	Reverse primer for site-directed mutagenesis of pACPM31 to produce a D575A replacement.	pACLK31-D
01-DacA	CAACATATGCAAGATTTTACCATTGCCGCTAAAC	Forward primer containing a *Nde*I site for cloning *dacA* from *S. pneumoniae* (residues 23 - 394) into pET-28a	pDSAC01
01-DacA	CGTTCTCGAGTCATTTTTCAATTTTCTTGTCTGCTACC	Reverse primer containing an *Xho*I site for cloning *dacA* from *S. pneumoniae* (residues 23 - 394) into pET-28a	pDSAC01
01-DacB	CAACATATGGAAGTGGTCAATAAAGGTGATTACTA	Forward primer containing a *Nde*I site for cloning *dacB* From *S. pneumoniae* (residues 56 - 238) into pET-28a.	pDSAC02
02-DacB	CGTTCTCGAGTTAATCGACGTAGTCTCCGCCTTC	Reverse primer containing an *Xho*I site for cloning *dacB* from *S. pneumoniae* (residues 56 - 238) into pET-28a.	pDSAC02
01-*Sp*OatA-A/S	CTGATCGGTGATGCGGTTGCTCTGCGT	Forward primer for site-directed mutagenesis of pDSAC81 to produce a S438A replacement.	pDSAC81-S438A
02-*Sp*OatA-A/S	ACGCAGAGCAACCGCATCACCGATCAG	Reverse primer for site-directed mutagenesis of pDSAC81 to produce a S438A replacement.	pDSAC81-S438A
01-*Sp*OatA-A/H	ACCGACCAAGTTGCCTTCGGTAGCGAA	Forward primer for site-directed mutagenesis of pDSAC81 to produce a H571A replacement.	pDSAC81-H571A
02-*Sp*OatA-A/H	TTCGCTACCGAAGGCAACTTGGTCGGT	Reverse primer for site-directed mutagenesis of pDSAC81 to produce a H571A replacement.	pDSAC81-H571A
01-*Sp*OatA-N/D	TGGGCGGGCACCAACCAAGTTCACTTC	Forward primer for site-directed mutagenesis of pDSAC81 to produce a D568Nn replacement.	pDSAC81-D568N
02-*Sp*OatA-N/D	GAAGTGAACTTGGTTGGTGCCCGCCCA	Reverse primer for site-directed mutagenesis of pDSAC81 to produce a D568N replacement.	pDSAC81-D568N
01-*Sp*OatA-N110A	GCCACGGGTGTCGCCAATCCGGAAAAC	Forward primer for site-directed mutagenesis of pDSAC81 to produce a N491A replacement.	pDSAC81-N491A
02-*Sp*OatA-N110A	GTTTTCCGGATTGGCGACACCCGTGGC	Reverse primer for site-directed mutagenesis of pDSAC81 to produce a N491A replacement.	pDSAC81-N491A
