# Supplementary figures and images for: In vitro characterization of the antivirulence target of Gram-positive pathogens, peptidoglycan O-acetyltransferase A (OatA)
Source: PLoS Pathog. 2017 Oct 27;13(10):e1006667. doi: 10.1371/journal.ppat.1006667 (PMC5697884; doi:10.1371/journal.ppat.1006667)

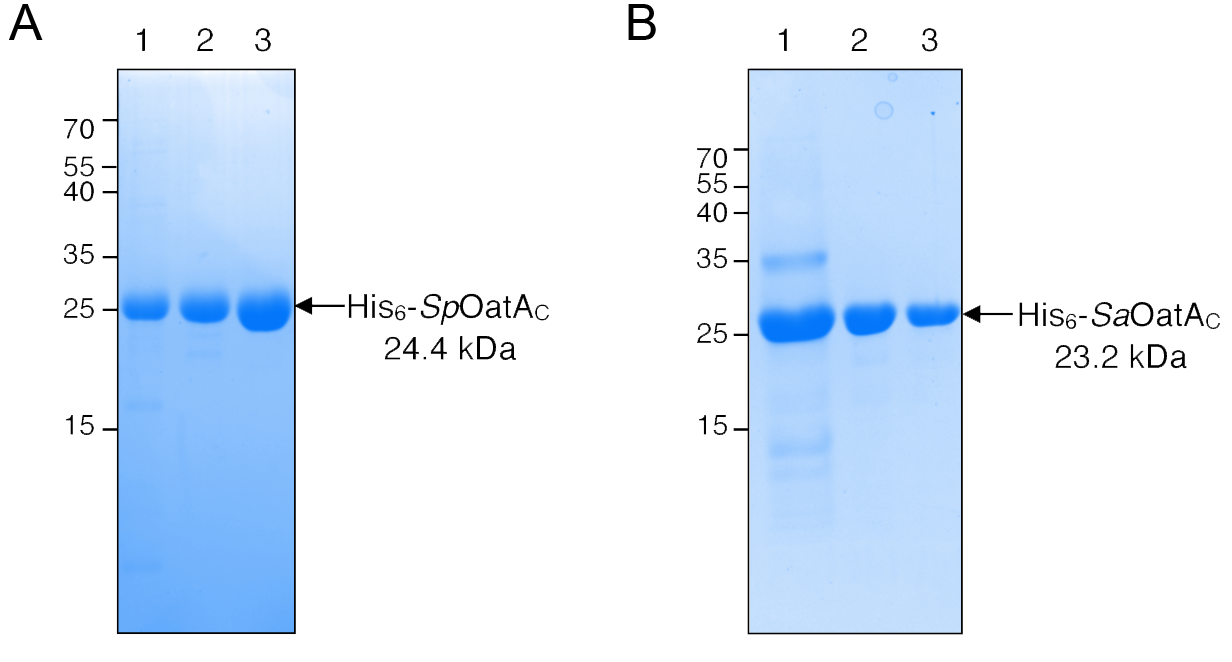

Supplement: S1 Fig — A. SDS-PAGE analysis of the purification of spOatAC. Lanes: 1, Elution fraction from His-Tag purification resin; 2 and 3, Fractions 1 and 3 of SourceQ elution, respectively. B. SDS-PAGE analysis of the purification of SaOatAC. Lanes 1, 2, and 3, correspond to the same purification steps as described in panel A. (TIF) [file ppat.1006667.s003.tif]

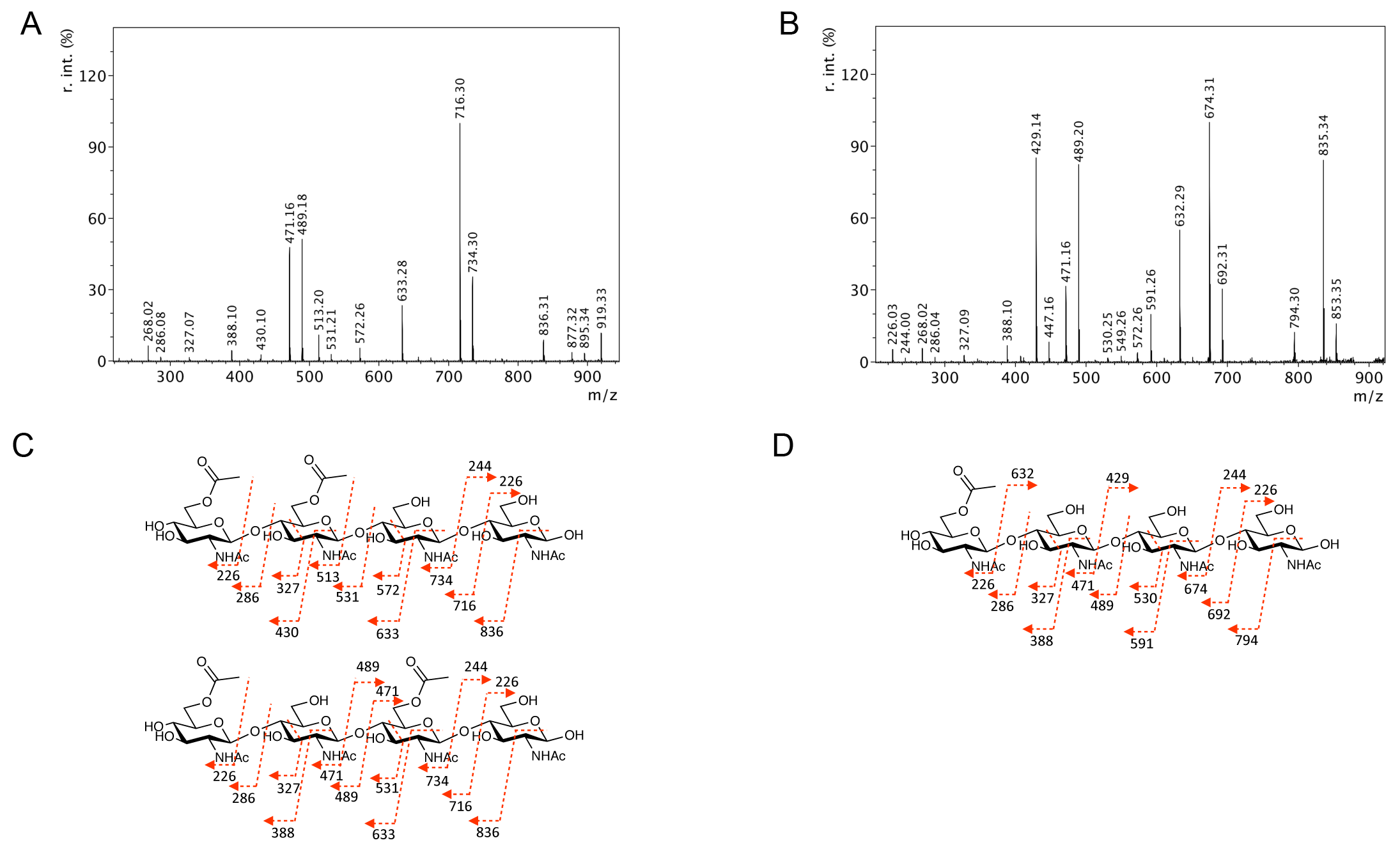

Supplement: S2 Fig — The MS/MS fragmentation spectra of A, the sodiated di-O-acetyl-chitotetraose parent ion (937.33 [M+Na]+) produced by SpOatAC and B, the sodiated O-acetyl-chitotetraose parent ion (895.35 [M+Na]+) produced by SaOatAC. Both sodium adducts were generated from the respective protonated species with 0.1 mM NaCl to facilitate the cross-ring cleavages. C and D, Interpretation of the fragment ions presented in panels A and B, respectively. (TIF) [file ppat.1006667.s004.tif]

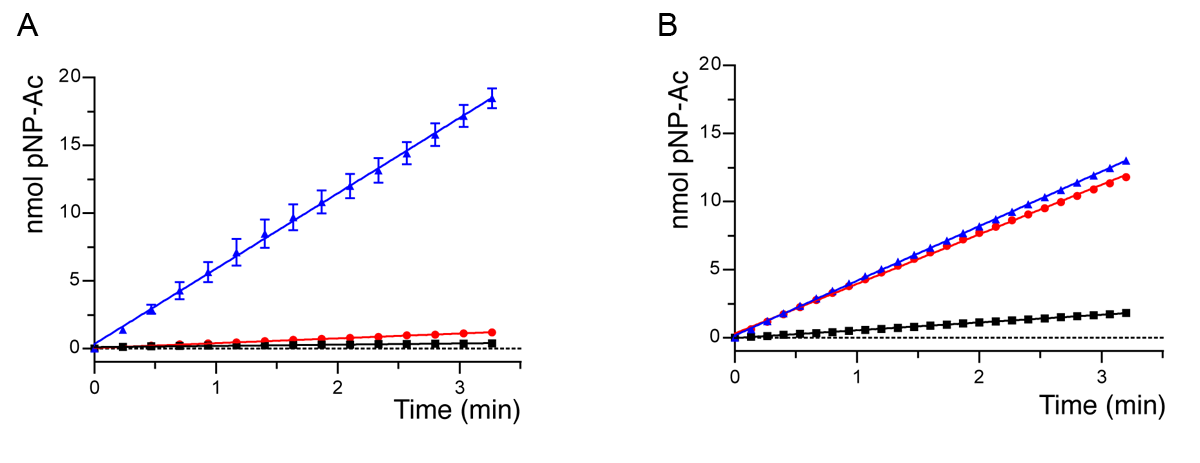

Supplement: S3 Fig — Progress curves of pNP release from 1 mM pNP-Ac in 50 mM sodium phosphate buffer pH 6.5 incubated at 25 ºC with A, SpOatAC and B, SaOatAC in the absence (red) and presence (blue) of 2 mM chitopentaose. The spontaneous release of pNP from pNP-Ac incubated under the same conditions but without added enzyme is represented by the black symbols. Each assay was performed in triplicate, with the s.e. noted. (TIF) [file ppat.1006667.s005.tif]

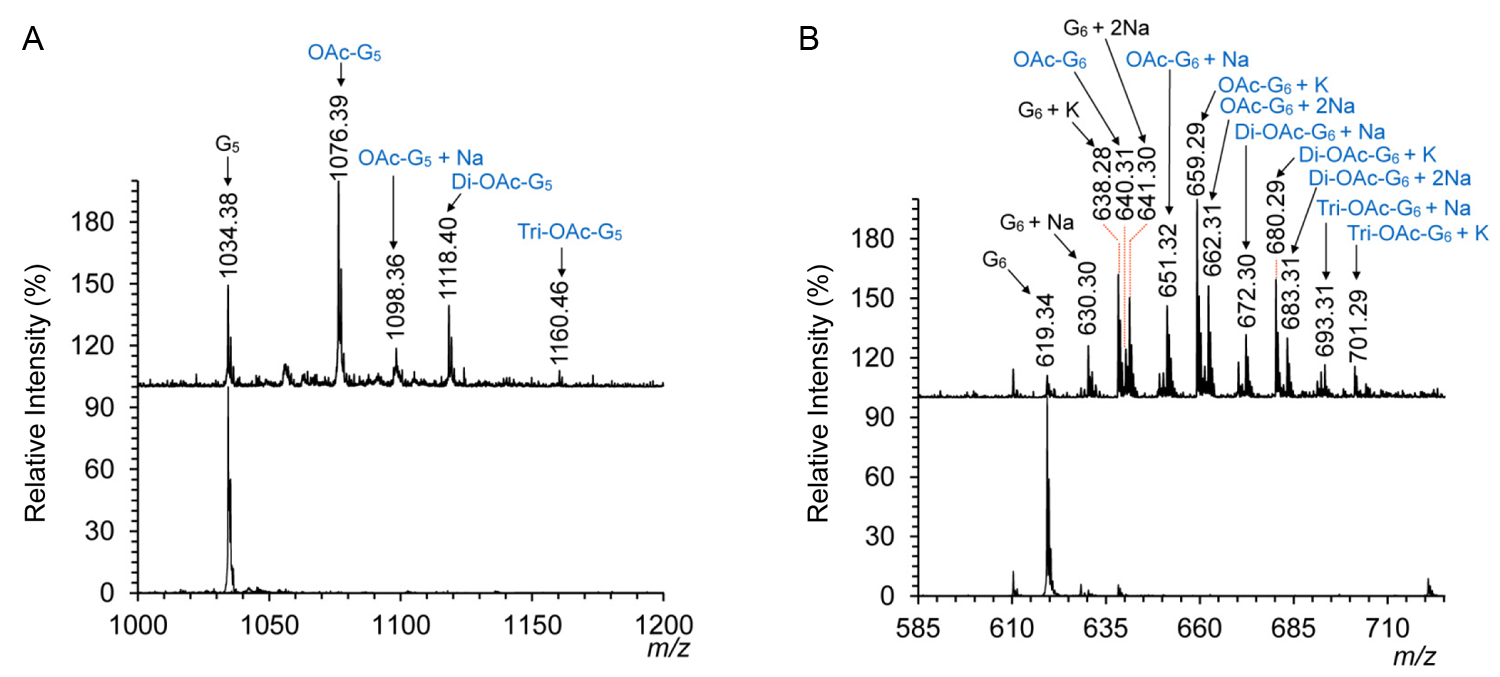

Supplement: S4 Fig — Enzyme (5 μM) in 50 mM sodium phosphate buffer pH 6.5 was incubated for 1 h at 37 ºC with 1 mM pNP-Ac and 2 mM A, chitopentaose (G5; [M+H/Na]+) and B, chitohexaose (G6; [M+H/Na/K]2+). Reaction products were isolated by adsorption to PGC solid-phase extraction cartridges prior to ESI-MS analysis 7by direct infusion using an Amazon SL ion-trap mass spectrometer. (TIF) [file ppat.1006667.s006.tif]

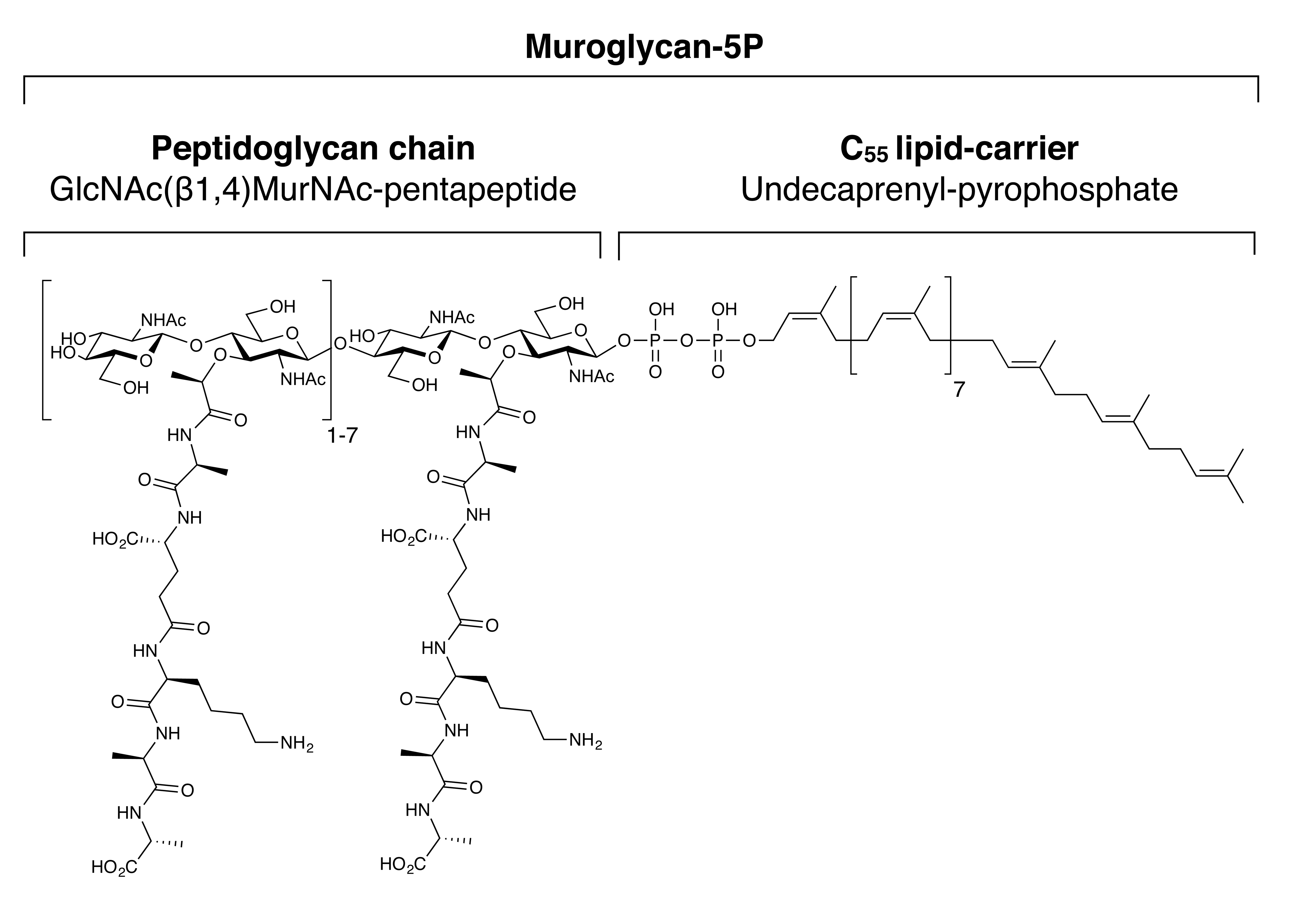

Supplement: S5 Fig — (TIF) [file ppat.1006667.s007.tif]

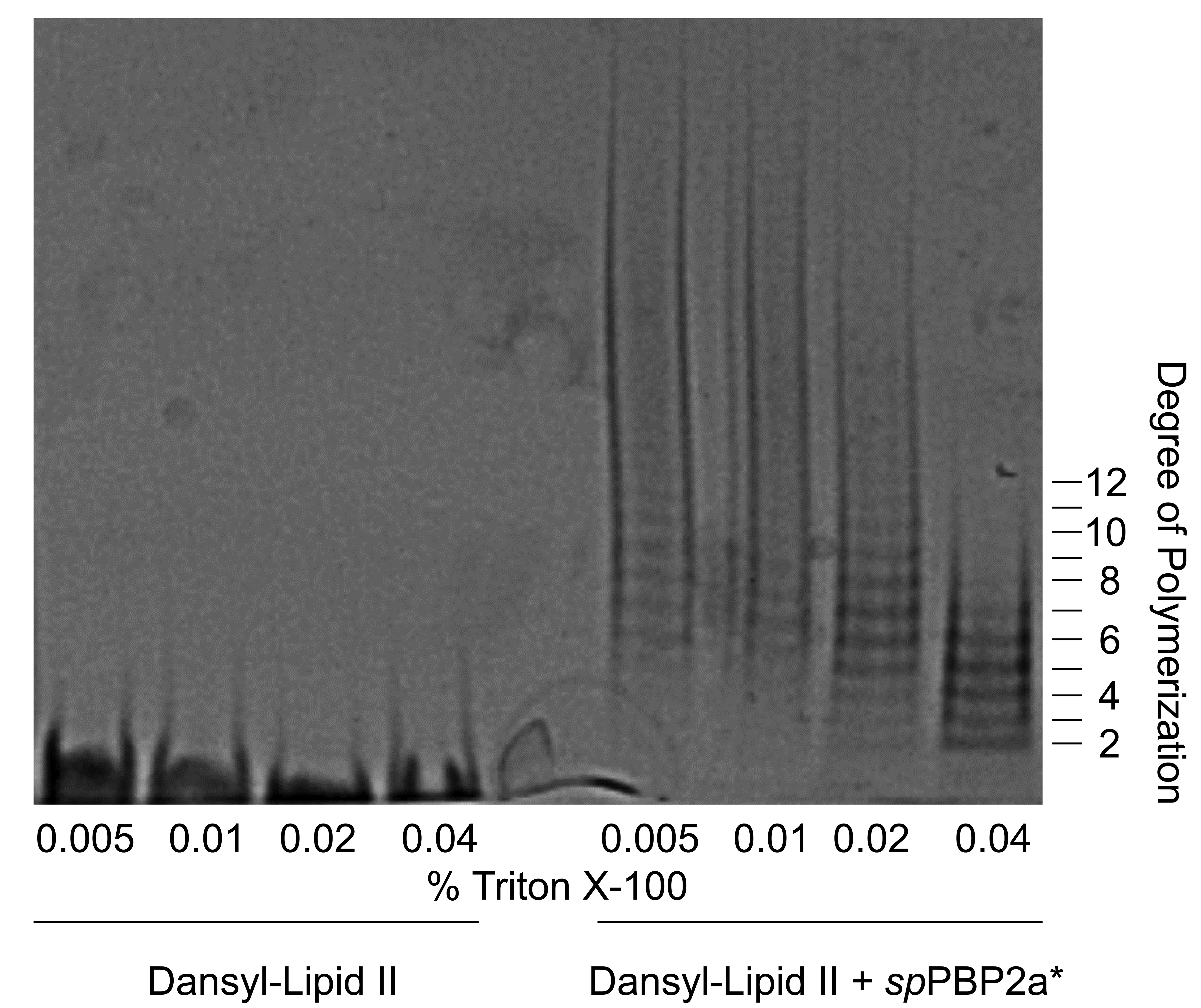

Supplement: S6 Fig — SDS PAGE analysis with fluorescence detection of reaction products of partially Dansylated lipid II (10 μM) in 50 mM HEPES buffer pH 7.5 containing 200 mM NaCl, 25 mM MgCl, 25% (v/v) DMSO, and varying concentrations of Triton X-100 as indicated in the absence and presence of incubated overnight at 30 ºC with S. pneumoniae PBP2a. (TIF) [file ppat.1006667.s008.tif]

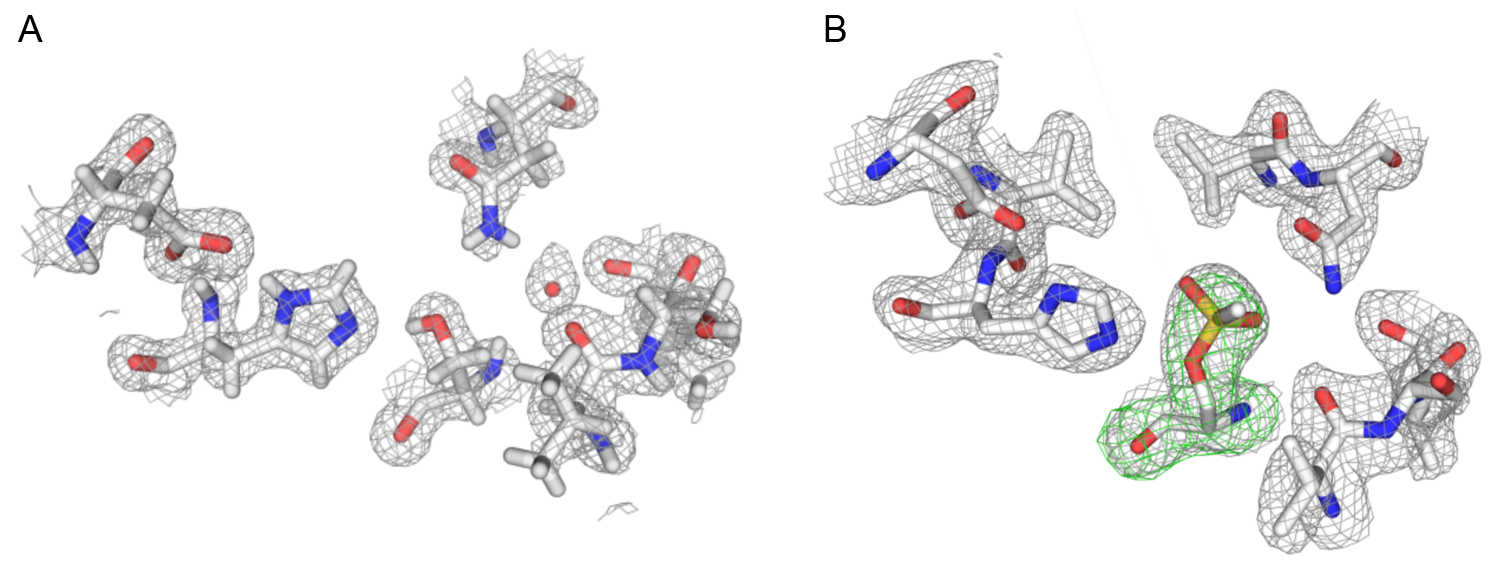

Supplement: S7 Fig — The 2mFo-D Fc maps (gray) of the active sites of A, native SpOatAC and B, SpOatAC-MeS are contoured at 1.0 σ. The mFo-D Fc omit map of the MeS-Ser438 adduct (green) is contoured at 3.0 σ. (TIF) [file ppat.1006667.s009.tif]

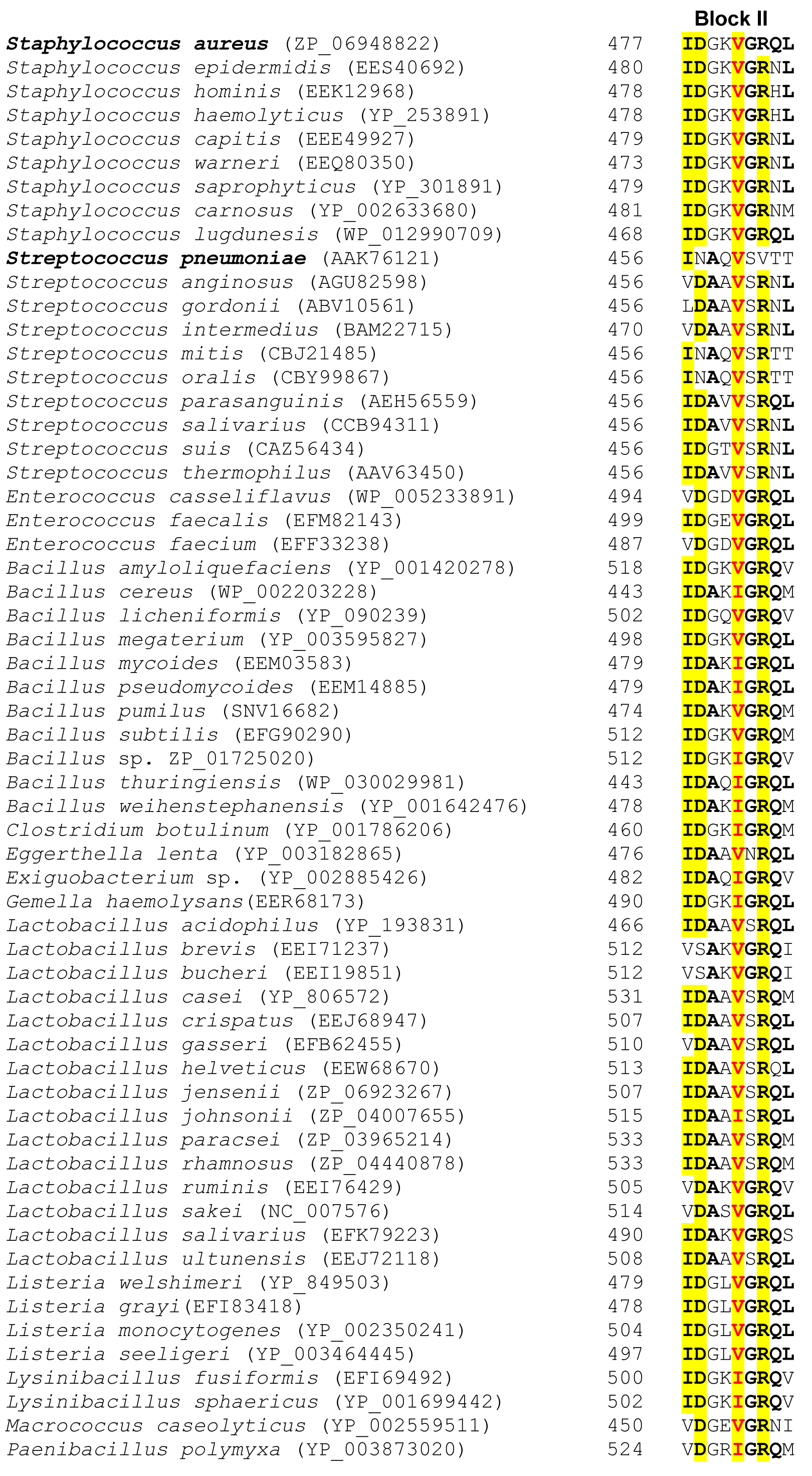

Supplement: S8 Fig — The sequence of S. aureus OatA was used as the query in a TBLASTN search of the completed bacterial genomes. The residues in bold face and highlighted in yellow denote greater than 50% and 80% identity, respectively, while invariant the Val/Ile at position 5 of the Block are in red. Only the sequence of a representative strain of the species listed is presented. (TIF) [file ppat.1006667.s010.tif]
